# Impact of donor stress-induced hyperglycemia on early graft outcomes in simultaneous pancreas-kidney transplantation: a retrospective cohort study

**DOI:** 10.3389/fimmu.2026.1783723

**Published:** 2026-06-12

**Authors:** Luhao Liu, Rongxin Chen, Yuhe Guo, Ziqing Guo, Jiao Wan, Guanghui Li, Jiali Fang, Junjie Ma, Zheng Chen

**Affiliations:** 1 Department of Organ Transplantation, The Second Affiliated Hospital, Guangzhou Medical University, Guangzhou, China; 2 Guangzhou University, Guangzhou, China; 3 General Surgery Department, Wangniudun Hospital, Dongguan, China

**Keywords:** donor selection, graft survival, organ donor, simultaneous pancreas-kidney transplantation, stress hyperglycemia

## Abstract

**Background:**

The suitability of donors with stress-induced hyperglycemia (SIH) for pancreas-containing transplants remains debated due to concerns over β-cell dysfunction. This study aimed to assess whether donor SIH is associated with early clinical graft outcomes, including metabolic recovery, complications, and graft/patient survival, following simultaneous pancreas-kidney transplantation (SPKT).

**Methods:**

In this retrospective cohort study, 251 SPKT recipients were stratified into two groups based on donor glycemic status during ICU stay: Normoglycemia (NG, n=41; all glucose measurements ≤11.1 mmol/L without insulin therapy) and SIH (n=210; sustained glucose >11.1 mmol/L requiring intravenous insulin infusion). Recipient demographics, postoperative pancreatic/renal graft function, complications, and long-term survival were compared between groups.

**Results:**

Donor and recipient baseline characteristics were comparable between groups, except for higher admission glucose in the SIH cohort. Postoperatively, no significant differences were observed between the SIH and NG groups in serial measurements of fasting glucose, HbA1c, C-peptide, serum amylase, or serum creatinine at 7 days, 1, 6, and 12 months (all p>0.05). Complication rates, including delayed graft function, acute rejection, graft thrombosis, and surgical fistulas, were also similar (all p>0.05). Most importantly, Kaplan-Meier analysis revealed no significant differences in death-censored pancreas or kidney graft survival, or in overall patient survival over the follow-up period (maximum 9.2 years) (all log-rank p>0.05).

**Conclusion:**

In this single-center, retrospective cohort with limited statistical power, we did not detect a statistically significant association between donor SIH and adverse early metabolic recovery, complication rates, or long-term graft and patient survival following SPKT. These observational data do not establish equivalence. The study is underpowered for uncommon events such as graft thrombosis, and clinically meaningful differences cannot be excluded. The findings should be interpreted as hypothesis-generating. They require prospective validation in larger, multi-center cohorts before informing clinical practice.

## Introduction

Simultaneous pancreas-kidney transplantation (SPKT) is the established gold standard for patients with type 1 diabetes and end-stage renal disease, offering the dual benefit of restoring physiological glycemic control and renal function, which results in superior long-term survival compared to kidney transplant alone or dialysis (1, 2). The procedure also confers multisystem advantages, including potential reversal of microvascular complications and reduced cardiovascular risk (3). Despite advances in surgical techniques and immunosuppression, a persistent global organ shortage limits access, with significant mortality on the waiting list. Although the appropriate expansion of donor selection criteria holds potential for greater benefit, definitive clinical evidence supporting this approach is currently lacking (4).

Stress-induced hyperglycemia (SIH), defined as transient glucose dysregulation (fasting glucose ≥6.9 mmol/L or random glucose ≥11.1 mmol/L) in critically ill patients without pre-existing diabetes, is a common yet insufficiently studied phenomenon in the intensive care unit (ICU) (5). The pathogenesis of SIH involves sympathetic overactivation, release of inflammatory cytokines (e.g., TNF-α, IL-6), and hypothalamic-pituitary-adrenal axis dysfunction, which collectively exacerbate insulin resistance and hepatic gluconeogenesis. Clinically, this metabolic dysregulation may predispose patients to an increased risk of organ failure or functional impairment (6). Although animal studies indicate that SIH induces reversible pancreatic oxidative stress, its clinical implications for pancreas donor viability and subsequent transplant outcomes remain uncertain (7).

Despite the absence of explicit contraindication in major guidelines, donors with SIH are frequently excluded from pancreas procurement in clinical practice. Neither the Eurotransplant P-PASS system nor OPTN/SRTR guidelines identify hyperglycemia or insulin use as independent exclusion criteria (8, 9). However, a national registry analysis demonstrated that donors receiving insulin in the ICU were significantly less likely to have their pancreas procured (OR 0.62, 95% CI 0.45–0.85), despite comparable post-transplant outcomes when utilized (10).

The physiological rationale for considering SIH donors is well-established. Hyperglycemia in brain-dead donors is common and multifactorial, arising from central nervous system injury, catecholamine release, and exogenous glucose and steroid administration, rather than reflecting intrinsic β-cell dysfunction (11, 12). In the absence of pre-existing diabetes, this hyperglycemia is reversible and does not necessarily indicate irreversible pancreatic damage (11). However, clinical studies examining the impact of donor SIH on outcomes in SPKT remain limited. The present study directly addresses this gap by evaluating the association between donor SIH and post-SPKT clinical outcomes, with the aim of informing donor selection policies, reducing unnecessary organ discard, and safely expanding the donor pool. This study does not investigate immunological mechanisms.

## Materials and methods

### Study design and participants

This retrospective cohort study utilized data from the SPKT database at the Second Affiliated Hospital of Guangzhou Medical University. A total of 251 patients who underwent SPKT between September 2016 and June 2024 were included. Donors were assigned to the SIH group if they met the following criterion during ICU stay: at least two consecutive blood glucose measurements >11.1 mmol/L within a 24-hour period. Insulin therapy was documented but was not a mandatory inclusion criterion for the SIH group. The normoglycemia (NG) group comprised donors with all glucose measurements ≤11.1 mmol/L and no insulin therapy. The threshold of 11.1 mmol/L was selected based on its established use as the diagnostic cutoff for diabetes mellitus and stress hyperglycemia in critical illness, reflecting clinically significant dysregulation beyond normal physiological variation. To further characterize glycemic status, we extracted detailed glucose data including peak glucose, number of hyperglycemic episodes, duration of hyperglycemia, insulin infusion rates, and HbA1c values when available.

To assess the robustness of our findings to alternative operationalizations of the exposure, we performed sensitivity analyses using three additional definitions of SIH: (A) peak glucose >11.1 mmol/L and insulin therapy; (B) peak glucose >11.1 mmol/L regardless of insulin therapy; and (C) at least three glucose measurements >11.1 mmol/L over a minimum of 24 hours. All primary outcome analyses were repeated using each definition.

Follow-up data were collected until December 1, 2025. Clinical parameters were collected and categorized into three domains: donor/recipient demographics (age, BMI, sex), graft function (pre- and postoperative fasting glucose, C-peptide, insulin, HbA1c, serum/urinary amylase, and serum creatinine), and postoperative complications (surgical, such as enteric or pancreatic fistula; and medical, including graft rejection, thrombosis, and infections).

### Ethical statement

This study was performed in accordance with the ethical principles of the Declaration of Helsinki and was approved by the Clinical Research and Application Ethics Committee of the Second Affiliated Hospital of Guangzhou Medical University (Approval No. 2022-hg-ks-17). Written informed consent was obtained from all transplant recipients. The authors attest to the integrity of the work and assume full responsibility for its content.

### Donor criteria

Potential donors were included for donor consideration in this cohort if they were aged 10 to 60 years with a BMI between 18 and 30 kg/m², and had no personal or family history of diabetes mellitus. Laboratory prerequisites consisted of a normal fasting C-peptide level (≥0.6 ng/mL) and a HbA1c value of less than 6.5%. Furthermore, donors were required to have no evidence of active malignancy and to exhibit normal pancreatic and renal anatomy on cross-sectional imaging (computed tomography or magnetic resonance imaging). In addition to standard donor and recipient characteristics, we collected the following donor variables specifically relevant to pancreas graft outcomes: serum amylase and lipase levels within 24 hours before procurement, daily insulin requirements during the final 24 hours of ICU stay, vasopressor support (type, number, and maximum dose), episodes of hemodynamic instability (defined as systolic blood pressure <90 mmHg for >30 minutes requiring fluid bolus or vasopressor escalation), and ICU length of stay. By design, all donors in this cohort had BMI between 18 and 30 kg/m²; therefore, no donors with underweight (BMI <18.5) or obesity (BMI ≥30) were included.

Donors were excluded in cases of direct pancreatic or renal trauma, death related to acute intoxication, the presence of uncontrolled multidrug-resistant infections, or a history of severe duodenal ulcers or relevant pancreatic/duodenal surgery.

### Recipient criteria

Eligible recipients were adults aged 18 to 65 years undergoing a first-time SPKT. Candidates were required to be free of active systemic infections, malignancies, and recent severe cardiovascular events at the time of evaluation. Exclusion criteria comprised transplants from living donors, procedures involving dual kidney or other multi-organ transplants, individuals with a history of prior kidney transplantation, and those undergoing re-transplantation of either organ.

### Surgical technique and postoperative monitoring

To minimize cold ischemia time, all pancreas and kidney grafts were procured from local donors within our hospital or from nearby hospitals within the same city, eliminating long-distance transport. Additionally, we employed a dual surgical team strategy: while one team performed the kidney transplant, the second team simultaneously prepared and back-table reconstructed the pancreas graft. This parallel workflow ensured that the pancreas graft was ready for implantation immediately upon completion of the kidney transplant, thereby reducing total cold ischemia time.

All patients underwent SPKT using a standardized unilateral approach on the right side, as previously described by our center (13, 14). The renal graft was placed in the retroperitoneum. The renal artery and vein were anastomosed end-to-side to the recipient’s external iliac artery and vein, respectively. Urinary reconstruction was performed via ureteroneocystostomy (or uretero-ureteral anastomosis, as applicable). The pancreatic graft was positioned intraperitoneally. For arterial reconstruction, the donor external iliac artery was anastomosed end-to-end to a Carrel patch containing the celiac axis and superior mesenteric artery of the graft. The donor common iliac artery was then anastomosed end-to-side to the recipient’s right external iliac artery. Venous outflow was established by anastomosing the graft portal vein end-to-side to the recipient’s vena cava. To optimize vascular perfusion and reduce the risk of thrombosis, we pay particular attention to preserving perfusion of the pancreatic tail after standard arterial and venous anastomoses: the donor splenic artery and vein are meticulously reconstructed to avoid thrombosis (**Supplementary Figure 1**). Exocrine drainage was accomplished via duodenal anastomosis to a loop of the recipient’s ileum (**Supplementary Video 1**).

To prevent graft thrombosis, all recipients receive standardized anticoagulation. Subcutaneous enoxaparin 2000-3000 IU twice daily is initiated 4-6 hours postoperatively. Doses are adjusted based on daily platelet count, and coagulation parameters. Anticoagulation is continued for at least 7 days; patients with additional thrombotic risk factors may receive extended prophylaxis for up to 2 weeks, transitioning to oral aspirin 100 mg daily.

All recipients undergo intensive postoperative monitoring for thrombosis. Color Doppler ultrasound is performed twice weekly for the first two weeks, then weekly until discharge, assessing renal and pancreatic graft vascular flow. Serum amylase, lipase, glucose, and creatinine are monitored daily. Any sudden increase in pancreatic enzymes or unexplained hyperglycemia prompts immediate Doppler ultrasound. Suspected thrombosis leads to urgent surgical exploration or interventional radiology consultation.

Exploratory histopathological analysis of time-zero pancreas biopsies was performed on a subset of donors. As these analyses were descriptive and not systematically quantified, the methods and findings are presented in **Supplementary Appendix 1**.

### Immunosuppression protocol

Induction regimens were stratified based on pre-transplant panel-reactive antibody (PRA) status. For PRA-negative recipients, therapy consisted of anti-thymocyte globulin (rabbit ATG 1 mg/kg/day or ATG 2 mg/kg/day, both for 5 days) combined with basiliximab (20 mg on day 0 and day 4). For PRA-positive recipients, the regimen comprised rabbit ATG (1 mg/kg/day for 4 days) plus rituximab (375 mg/m², administered as two doses).

Maintenance immunosuppression comprised a triple-drug regimen: tacrolimus (initial dose 0.05-0.10 mg/kg/day, with a target trough level of 8-10 ng/mL), mycophenolate mofetil (1.0-1.5 g/day, targeting an AUC of 30-60 mg·h/L), and prednisone. Prednisone was initiated at 25 mg/day and subsequently tapered to a maintenance dose of 5 mg/day.

### Statistical analysis

Statistical analyses were performed using SPSS version 25.0 and R version 4.2.1. A two-sided P < 0.05 was considered statistically significant.

### Primary analyses

Normally distributed continuous variables are presented as mean ± SD and compared using Student’s t-test; non-normally distributed data are expressed as median (IQR) and compared using the Mann-Whitney U test. Categorical variables are summarized as number (%) and analyzed using the chi-square or Fisher’s exact test, as appropriate.

Longitudinal outcomes (fasting glucose, HbA1c, C-peptide, insulin, serum amylase, serum creatinine) were analyzed using linear mixed models for repeated measures. Each model included group (SIH vs. NG), time (postoperative day 7, 1 month, 6 months, 1 year), and their interaction as fixed effects, with a random intercept for each subject and an unstructured covariance matrix. Model-estimated means with 95% CIs are presented. A two-sided P < 0.05 was considered statistically significant.

Graft and patient survival were estimated by the Kaplan-Meier method and compared using the log-rank test. Risk factors were evaluated using Cox proportional hazards regression. Variables with P<0.10 in univariable analysis or clinical relevance were entered into multivariable models, including donor age, donor BMI, donor hypertension, cold ischemia time, recipient age, recipient BMI, dialysis duration, HLA mismatch, and donor SIH status. Results are presented as hazard ratios (HR) with 95% confidence intervals (CI). The proportional hazards assumption was verified by Schoenfeld residuals (all P > 0.05) and visual inspection of log-minus-log plots. To account for death as a competing risk, the Fine-Gray subdistribution hazard model was applied, adjusting for the same covariates; results are reported as subdistribution HRs with 95% CIs.

### Sensitivity analysis: propensity score matching

To address the imbalance in group size and potential confounding, we performed propensity score matching (PSM) as a sensitivity analysis. Propensity scores were estimated using logistic regression based on clinically relevant covariates that could influence both donor glycemic status and transplant outcomes: donor age, donor BMI, donor hypertension, cause of brain death, cold ischemia time, recipient age, recipient BMI, dialysis duration, and HLA mismatch. SIH and NG donors were matched 1:1 using nearest-neighbor matching without replacement, with a caliper width of 0.2 times the standard deviation of the logit propensity score. Covariate balance before and after matching was assessed using standardized mean differences (SMD), with values < 0.1 indicating adequate balance. All primary outcome analyses (linear mixed models for functional outcomes, Kaplan-Meier survival analysis, Cox regression, and competing risk models) were subsequently repeated in the propensity score-matched cohort to validate the robustness of our findings. Results of these sensitivity analyses are presented in the **Supplementary Materials**.

## Results

### Baseline characteristics

This study included 251 recipients who underwent SPKT, categorized into a SIH group (n = 210) and a NG group (n = 41). All 251 patients (100%) were included in the survival analyses. For postoperative functional assessments, follow-up rates were 100% at postoperative day 7 (n=251), 98.4% at 1 month (n=247), 91.2% at 6 months (n=229), and 85.3% at 1 year (n=214). Recipient demographics and clinical profiles are presented in **Table 1**. There were no significant differences between the SIH and NG groups in terms of age, BMI, gender, dialysis duration, primary renal disease, prevalence of hypertension, distribution of diabetes types, PRA status, HLA mismatch, or dialysis modality (all P > 0.05). As shown in **Table 2**, donors in the SIH group had significantly higher admission blood glucose levels compared to the NG group (median [IQR]: 9.24 [6.85-12.40] mmol/L vs. 6.23 [4.78-8.08] mmol/L, P < 0.001). Other donor characteristics, including age, BMI, sex distribution, pre-procurement serum creatinine, cause of brain death, history of hypertension, warm ischemia time, cold ischemia time, and total transplant surgery time, were comparable between the two groups (all P > 0.05). Comparison of additional donor characteristics between the SIH and NG groups is presented in **Supplementary Table 1**. Aside from insulin requirements (which were part of the SIH definition), no significant differences were observed between groups for donor amylase, lipase, vasopressor support, hemodynamic instability, or ICU length of stay (all P > 0.05).

**Table 1:** General characteristics of recipients in the SIH and NG group.

Basic characteristics	SIH group (n=210)	NG group (n=41)	P value
Age (years)	49.4 ± 9.6	50 ± 11.1	0.719
BMI (kg/m²)	23.7 ± 3.1	23.8 ± 2.9	0.827
Gender			0.565
Male	177 (84.3%)	36 (87.8%)	
Female	33 (15.7%)	5 (12.2%)	
Dialysis Duration (months)	14.6 ± 16.8	12.1 ± 12.4	0.372
Primary Disease			0.594
Diabetic Nephropathy	172 (81.9%)	35 (85.4%)	
Chronic Glomerulonephritis	38 (18.1%)	6 (14.6%)	
Hypertension			0.968
Yes	195 (92.9%)	38 (92.7%)	
No	15 (7.1%)	3 (7.3%)	
Types of diabetes			0.886
Type 1	19 (9.1%)	4 (9.8%)	
Type 2	191 (90.9%)	37 (90.2%)	
PRA			0.438
Positive	30 (14.3%)	4 (9.8%)	
Negative	180 (85.7%)	37 (90.2%)	
HLA Mismatch			0.893
1	18 (8.6%)	5 (12.2%)	
2	44 (20.9%)	9 (21.9%)	
3	109 (51.9%)	20 (48.8%)	
4	39 (18.6%)	7 (17.1%)	
Dialysis Type			0.379
No Dialysis	12 (5.7%)	2 (4.9%)	
Peritoneal Dialysis	18 (8.6%)	1 (2.4%)	
Hemodialysis	180 (85.7%)	38 (92.7%)	

Data are presented as mean ± SD or n (%). BMI, body mass index; PRA, panel-reactive antibody; HLA, human leukocyte antigen.

**Table 2:** General characteristics of donors in the SIH and NG group.

Donor characteristics	SIH group (n=210)	NG group (n=41)	P value
Age (years)	34.68 ± 12.25	32.68 ± 10.87	0.332
Admission blood glucose (mmol/L)	9.24 (6.85-12.40)	6.23 (4.78-8.08)	<0.001
BMI (kg/m²)	22.8 ± 3.48	22.39 ± 3.24	0.490
Pre-procurement serum creatinine (μmol/L)	152.9 ± 115.6	120.3 ± 88.4	0.089
Cause of Brain Death			0.925
Traumatic Brain Injury	124 (59%)	24 (58.5%)	
Cerebral Hemorrhage	68 (32.4%)	14 (34.2%)	
Other Causes	18 (8.6%)	3 (7.3%)	
Hypertension			0.433
Yes	36 (17.1%)	5 (12.2%)	
No	174 (82.9%)	36 (87.8%)	
Ischemia-Reperfusion Injury			
Warm Ischemia Time (min)	1.01 ± 1.13	1.02 ± 1.48	0.103
Cold Ischemia Time (h)	3.45 ± 1.92	3.28 ± 1.49	0.290
Transplant Surgery Time (h)	6.18 ± 0.34	6.19 ± 0.29	0.844

Data are presented as mean ± SD, median (IQR), or n (%). SIH, stress-induced hyperglycemia; NG, normoglycemia. BMI, body mass index.

Notably, the majority of recipients in both groups had type 2 diabetes (90.9% in the SIH group vs. 90.2% in the NG group). This distribution reflects the substantial absolute number of type 2 diabetic patients with end-stage renal disease in China-a consequence of the enormous type 2 diabetic population base (approximately 90-95% of all diabetes cases) (15) and the high overall prevalence of diabetes (exceeding 11% of adults) (16). It is important to note that while the risk of developing end-stage renal disease is higher among individuals with type 1 diabetes, the vastly larger type 2 diabetic population results in a greater absolute number of type 2 diabetic patients requiring renal replacement therapy. This pattern is consistent with our center’s previously reported experience of offering SPKT to carefully selected patients with type 2 diabetes and favorable metabolic profiles (17).

In univariable analysis (**Supplementary Table 2**), none of these additional donor variables were significantly associated with pancreas graft failure (all P > 0.05). Expanded multivariable Cox models incorporating all additional variables confirmed that donor SIH remained non-significant (HR = 1.16, 95% CI: 0.58–2.32, P = 0.678), and none of the additional variables were retained as independent predictors in stepwise selection. Donor age >45 years remained the only significant independent risk factor (HR = 2.28, 95% CI: 1.24–4.19, P = 0.008).

### Donor glycemic characteristics

Detailed glycemic profiles of donors are presented in **Table 3**. Compared to the NG group, donors in the SIH group had significantly higher peak glucose levels (14.8 ± 3.2 vs. 8.9 ± 1.6 mmol/L, P < 0.001), more frequent hyperglycemic episodes [median 4 (IQR 2-7) vs. 0], and longer duration of hyperglycemia [median 18 (IQR 8-36) vs. 0 hours]. Insulin therapy was administered to 88.1% of SIH donors, with a median maximum infusion rate of 2.5 units/hour. HbA1c levels were comparable between groups (5.4 ± 0.4 vs. 5.3 ± 0.3%, P = 0.182), confirming the absence of pre-existing diabetes.

**Table 3:** Detailed donor glycemic profiles.

Parameter	SIH group (n=210)	NG group (n=41)	P value
Admission glucose (mmol/L), median (IQR)	9.24 (6.85-12.40)	6.23 (4.78-8.08)	<0.001
Peak glucose during ICU stay (mmol/L), mean ± SD	14.8 ± 3.2	8.9 ± 1.6	<0.001
Number of glucose measurements >11.1 mmol/L, median (IQR)	4 (2-7)	0 (0-0)	<0.001
Duration of hyperglycemia >11.1 mmol/L (hours), median (IQR)	18 (8-36)	0 (0-0)	<0.001
Insulin therapy administered, n (%)	185 (88.1%)	0 (0%)	<0.001
Maximum insulin infusion rate (units/hour), median (IQR)	2.5 (1.0-4.5)	0 (0-0)	<0.001
Duration of insulin therapy (hours), median (IQR)	24 (12-48)	0 (0-0)	<0.001
HbA1c (%), mean ± SD	5.4 ± 0.4	5.3 ± 0.3	0.182
Last glucose before procurement (mmol/L), mean ± SD	8.2 ± 2.4	6.1 ± 1.2	<0.001

Insulin therapy was administered to 88.1% of SIH donors but was not a mandatory inclusion criterion for the SIH group. The decision to initiate insulin was made according to local ICU protocols and clinical circumstances, including timing of procurement and hemodynamic stability.

Abbreviations: SIH, stress-induced hyperglycemia; NG, normoglycemia; IQR, interquartile range; SD, standard deviation; ICU, intensive care unit; HbA1c, hemoglobin A1c.

Sensitivity analyses using three alternative definitions of SIH yielded results consistent with the primary analysis. Regardless of the definition used, donor SIH was not associated with differences in longitudinal graft function, postoperative complications, or graft and patient survival (**Supplementary Tables 3–5**). These findings indicate that the results were consistent across alternative definitions of the exposure.

Longitudinal Analysis of Pancreatic and Renal Graft Function After SPKT Linear mixed models for repeated measures were used to compare the trajectory of graft function between the SIH and NG groups over the 1-year follow-up period (**Tables 4**, **5**). For all pancreatic function parameters-including fasting glucose, HbA1c, C-peptide, and serum amylase-there were no significant group-by-time interactions (all P > 0.05) and no significant main effects of group (all P > 0.05), indicating comparable trajectories of functional recovery over time between groups; there was also no evidence of an overall difference between groups. For fasting insulin, although a transient difference was observed at day 7 in the unadjusted analysis, the linear mixed model revealed no significant group effect (P = 0.342) or group-by-time interaction (P = 0.287). Similarly, for serum creatinine, no significant group effect (P = 0.683) or interaction effect (P = 0.791) was detected, confirming equivalent renal function recovery between groups throughout the follow-up period. It should be noted that given the multiple comparisons performed across numerous outcomes and time points, this isolated significant finding at a single early time point should be interpreted with caution, as it may represent a chance observation rather than a true biological effect.

**Table 4:** Longitudinal analysis of pancreatic graft function: model-estimated means (95% CI).

Outcome	Time point	SIH group	NG group	P value (group)	P value (interaction)
Fasting Glucose (mmol/L)				0.452	0.381
	Postop day 7	7.62 (7.18–8.06) (n=210)	7.51 (6.89–8.13) (n=41)		
	1 month	6.02 (5.68–6.36) (n=206)	6.19 (5.67–6.71) (n=41)		
	6 months	5.35 (5.09–5.61) (n=192)	5.64 (5.22–6.06) (n=37)		
	1 year	5.43 (5.17–5.69) (n=180)	5.81 (5.39–6.23) (n=34)		
HbA1c (%)				0.612	0.543
	Postop day 7	6.54 (6.32–6.76) (n=210)	6.48 (6.14–6.82) (n=41)		
	1 month	6.14 (5.96–6.32) (n=207)	6.12 (5.84–6.40) (n=41)		
	6 months	5.71 (5.57–5.85) (n=192)	5.73 (5.51–5.95) (n=37)		
	1 year	5.70 (5.56–5.84) (n=181)	5.75 (5.53–5.97) (n=34)		
Fasting C-peptide (ng/mL)				0.527	0.612
	Postop day 7	9.58 (8.79–10.37) (n=210)	9.12 (7.93–10.31) (n=41)		
	1 month	7.28 (6.67–7.89) (n=207)	6.91 (6.01–7.81) (n=41)		
	6 months	5.12 (4.66–5.58) (n=193)	4.95 (4.27–5.63) (n=37)		
	1 year	4.78 (4.39–5.17) (n=181)	4.86 (4.27–5.45) (n=34)		
Fasting Insulin (μIU/mL)				0.342	0.287
	Postop day 7	22.89 (20.43–25.35) (n=210)	28.64 (24.86–32.42) (n=41)		
	1 month	18.76 (17.01–20.51) (n=208)	18.59 (15.80–21.38) (n=40)		
	6 months	15.91 (14.56–17.26) (n=193)	16.24 (14.15–18.33) (n=37)		
	1 year	15.62 (14.30–16.94) (n=181)	17.28 (15.23–19.33) (n=34)		
Serum Amylase (U/L)				0.589	0.468
	Postop day 7	201.2 (175.3–227.1) (n=210)	214.9 (175.8–254.0) (n=41)		
	1 month	158.9 (142.1–175.7) (n=208)	142.8 (118.1–167.5) (n=41)		
	6 months	108.3 (97.1–119.5) (n=192)	106.2 (89.7–122.7) (n=37)		
	1 year	93.8 (84.6–103.0) (n=181)	100.5 (86.4–114.6) (n=34)		

*Data are presented as model-estimated means (95% CI) from linear mixed models for repeated measures. Sample sizes at each time point are shown in parentheses. SIH, stress-induced hyperglycemia; NG, normoglycemia; HbA1c, hemoglobin A1c.*.

**Table 5:** Longitudinal analysis of renal graft function: model-estimated means (95% CI).

Time point	SIH group	NG group	P value (group)	P value (interaction)
Preoperative	832.4 (785.6-879.2) (n=210)	841.7 (773.2-910.2) (n=41)	0.767¹	–
Postop day 7	187.6 (165.2-210.0) (n=210)	168.4 (135.7-201.1) (n=41)	0.301	0.791
1 month	136.8 (126.3-147.3) (n=207)	129.5 (114.0-145.0) (n=41)		
6 months	133.2 (124.1-142.3) (n=191)	130.8 (117.5-144.1) (n=37)		
1 year	136.1 (127.2-145.0) (n=180)	135.9 (122.8-149.0) (n=34)		

Data are presented as model-estimated means (95% CI) in μmol/L from linear mixed models for repeated measures. Sample sizes at each time point are shown in parentheses. SIH, stress-induced hyperglycemia; NG, normoglycemia.

¹P value for preoperative comparison from t-test.

### Comparison of postoperative complications

The incidence of postoperative complications was compared between the SIH and NG groups, as detailed in **Table 6**. No statistically significant differences were observed in the rates of major transplant-related complications between the two groups. Specifically, the incidence of delayed graft function was 6.2% in the SIH group versus 7.3% in the NG group (P = 0.787). Acute rejection rates were also comparable, with kidney rejection occurring in 8.6% vs. 7.3% (P = 0.791) and pancreas rejection in 7.6% vs. 4.9% (P = 0.534), respectively. Similarly, the frequencies of surgical complications-including enteric fistula (1.9% vs. 4.9%, P = 0.254), pancreatic fistula (0.9% vs. 2.4%, P = 0.423), pancreatic graft thrombosis (5.7% vs. 7.3%, P = 0.456), intestinal tract bleeding (10% vs. 14.6%, P = 0.381), and intestinal obstruction (3.3% vs. 7.3%, P = 0.233) did not differ significantly between the SIH and NG groups.

**Table 6:** Comparison of complications between the SIH and NG group.

Factors	SIH group (n=210)	NG group (n=41)	P value
DGF	13 (6.2%)	3 (7.3%)	0.787
Kidney Rejection	18 (8.6%)	3 (7.3%)	0.791
Pancreas Rejection	16 (7.6%)	2 (4.9%)	0.534
Enteric Fistula	4 (1.9%)	2 (4.9%)	0.254
Pancreatic Fistula	2 (0.9%)	1 (2.4%)	0.423
Pancreatic Graft Thrombosis	12 (5.7%)	3 (7.3%)	0.456
Intestinal tract bleeding	21 (10%)	6 (14.6%)	0.381
Intestinal obstruction	7 (3.3%)	3 (7.3%)	0.233

Data are presented as n (%). SIH, stress-induced hyperglycemia; NG, normoglycemia. DGF, delayed graft function.

### Comparison of survival outcomes

**Figure 1:**
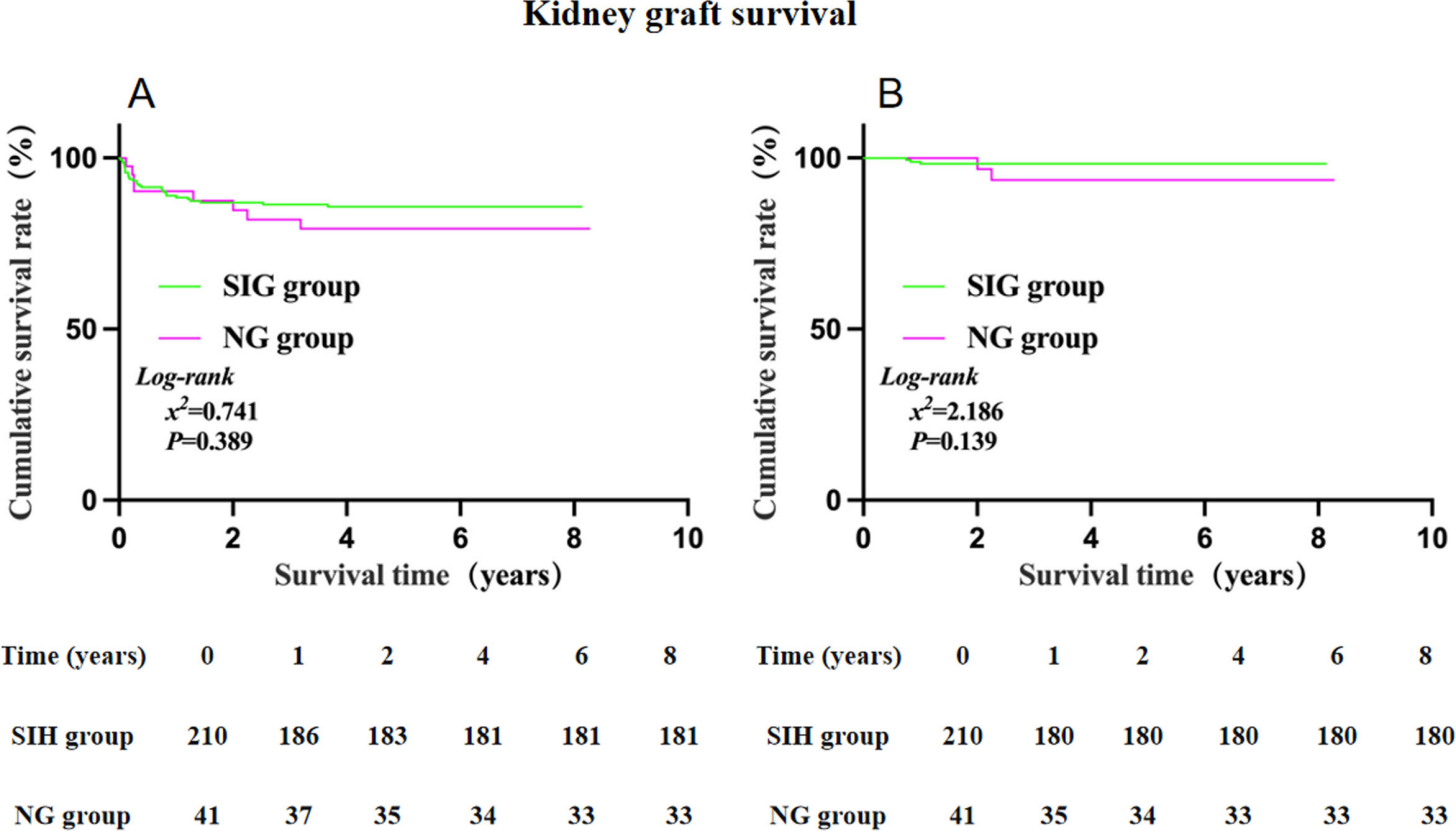
Kidney graft survival outcomes following simultaneous pancreas-kidney transplantation (SPKT). **(A)** Kaplan-Meier curves for kidney graft survival in recipients of grafts from donors with stress-induced hyperglycemia (SIH group, blue line) versus normoglycemic donors (NG group, red line). No significant difference was observed (Log-rank χ² = 0.741, P = 0.389). The number of patients at risk at each time point is shown below the x−axis. **(B)** Kaplan-Meier curves for death-censored kidney graft survival (censoring patient death with a functioning graft) in the SIH and NG groups. Survival between the two groups was comparable (Log-rank χ² = 2.186, P = 0.139). The number of patients at risk at each time point is shown below the x−axis.

**Figure 2:**
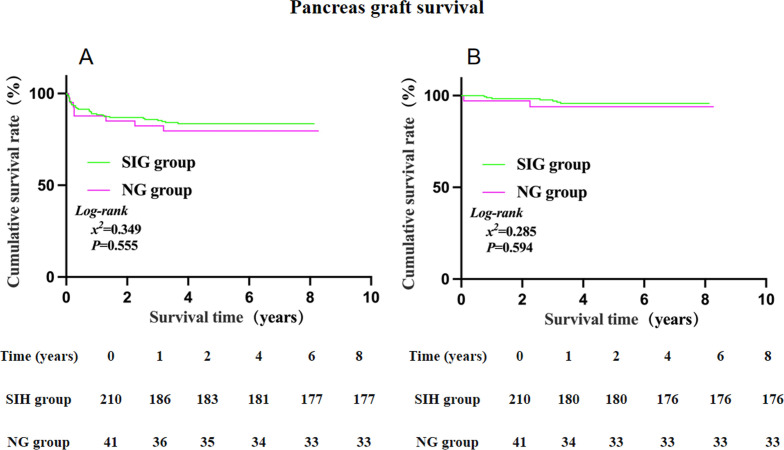
Pancreas graft survival outcomes following simultaneous pancreas-kidney transplantation (SPKT). **(A)** Kaplan-Meier curves for pancreas graft survival in the SIH and NG groups. The difference was not statistically significant (Log-rank χ² = 0.349, P = 0.555). The number of patients at risk at each time point is shown below the x−axis. **(B)** Kaplan-Meier curves for death-censored pancreas graft survival in both groups, showing no significant difference (Log-rank χ² = 0.285, P = 0.594). The number of patients at risk at each time point is shown below the x−axis.

**Figure 3:**
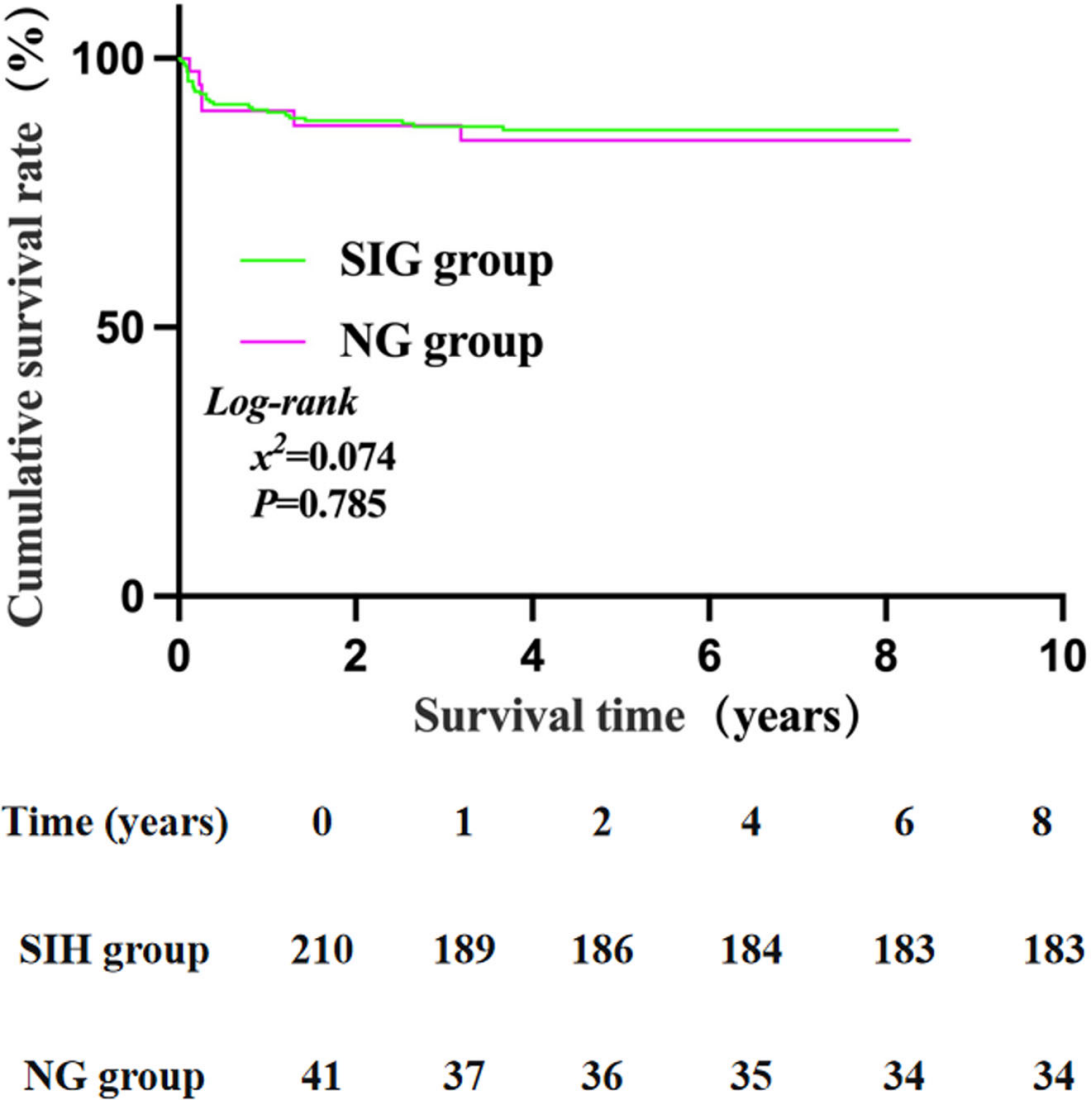
Overall patient survival following simultaneous pancreas-kidney transplantation (SPKT). Kaplan-Meier curves comparing overall patient survival between recipients in the SIH and NG groups throughout the follow-up period (maximum 9.2 years). No significant difference was found (Log-rank χ² = 0.074, P = 0.785). The number of patients at risk at each time point is shown below the x−axis.

Long-term survival outcomes did not differ significantly between the SIH and NG groups. Kidney graft survival (**Figure 1A**) and death-censored kidney graft survival (**Figure 1B**) showed no significant differences (Log-rank χ² = 0.741, P = 0.389 and Log-rank χ² = 2.186, P = 0.139, respectively). Similarly, pancreas graft survival (**Figure 2A**) and death-censored pancreas graft survival (**Figure 2B**) did not differ significantly between groups (Log-rank χ² = 0.349, P = 0.555 and Log-rank χ² = 0.285, P = 0.594, respectively). Overall patient survival throughout the follow-up period (maximum 9.2 years) was also equivalent between the two groups (Log-rank χ² = 0.074, P = 0.785; **Figure 3**).

Multivariable Cox regression analysis, adjusting for donor age, donor BMI, donor hypertension, cold ischemia time, recipient age, recipient BMI, dialysis duration, HLA mismatch, and donor SIH status, did not identify donor SIH status as a statistically significant independent predictor of death-censored kidney graft failure (HR = 1.24, 95% CI: 0.68-2.26, P = 0.482), death-censored pancreas graft failure (HR = 1.18, 95% CI: 0.62-2.25, P = 0.618), or patient death (HR = 0.92, 95% CI: 0.45-1.88, P = 0.821). The only independent risk factor identified was donor age >45 years for pancreas graft failure (HR = 2.31, 95% CI: 1.28-4.17, P = 0.006). The full multivariable models, including all covariates with their hazard ratios and confidence intervals, are presented in **Supplementary Table 10**. A competing-risk analysis using the Fine-Gray subdistribution hazard model, which accounts for death as a competing event, was conducted to validate the primary survival findings. After adjusting for the aforementioned covariates, results remained unchanged; donor SIH status was not independently associated with kidney graft failure accounting for death as a competing risk (SHR = 1.24, 95% CI: 0.68-2.26, P = 0.482) or pancreas graft failure accounting for death as a competing risk (SHR = 1.18, 95% CI: 0.62-2.25, P = 0.618) (**Supplementary Figure 2**). These results do not provide evidence of a statistically significant association between donor SIH and graft or patient survival in this cohort; however, the confidence intervals are wide and do not exclude clinically meaningful effects.

### Sensitivity analysis using propensity score matching

To address the potential confounding and imbalance in group sizes, we performed a PSM analysis as described in the Methods. From the original cohort of 210 SIH donors and 41 NG donors, we successfully matched 41 SIH donors to 41 NG donors using 1:1 nearest-neighbor matching without replacement (caliper width 0.2×SD of logit propensity score). The propensity score model included donor age, donor BMI, donor hypertension, cause of brain death, cold ischemia time, recipient age, recipient BMI, dialysis duration, and HLA mismatch.

After matching, all covariates were well-balanced between the two groups, with SMD < 0.1 for all variables (**Supplementary Table 6**). Baseline characteristics of the matched cohort are presented in **Supplementary Table 7**; no significant differences were observed between the SIH and NG groups in any donor or recipient characteristic, confirming the adequacy of the matching process.

### Longitudinal graft function in the matched cohort

Linear mixed models for repeated measures were reapplied to the matched cohort for all functional outcomes (fasting glucose, HbA1c, C-peptide, insulin, serum amylase, and serum creatinine). Consistent with the primary analysis, there were no significant group effects or group-by-time interactions for any parameter (all P > 0.05), indicating comparable trajectories of pancreatic and renal function recovery between SIH and NG donors (**Supplementary Table 8**).

### Postoperative complications in the matched cohort

The incidence of postoperative complications, including delayed graft function, acute rejection, graft thrombosis, and surgical fistulas, did not differ significantly between the SIH and NG groups in the matched cohort (all P > 0.05; **Supplementary Table 9**).

### Graft and patient survival in the matched cohort

Kaplan-Meier analysis in the matched cohort revealed no significant differences between the SIH and NG groups in kidney graft survival (log-rank P = 0.412), death-censored kidney graft survival (log-rank P = 0.508), pancreas graft survival (log-rank P = 0.538), death-censored pancreas graft survival (log-rank P = 0.624), or overall patient survival (log-rank P = 0.671). Multivariable Cox regression adjusting for the matching covariates confirmed that donor SIH status was not independently associated with death-censored kidney graft failure (HR = 1.12, 95% CI: 0.51–2.46, P = 0.779) or death-censored pancreas graft failure (HR = 1.08, 95% CI: 0.48–2.43, P = 0.852). Competing risk analysis using the Fine-Gray model, accounting for death as a competing event, similarly showed no association between donor SIH and kidney graft failure (SHR = 1.09, 95% CI: 0.49–2.42, P = 0.831) or pancreas graft failure (SHR = 1.04, 95% CI: 0.46–2.35, P = 0.925) (**Supplementary Figure 3**).

### Summary of sensitivity analysis

All results from the propensity score-matched cohort were consistent with those of the primary analysis, demonstrating that donor SIH was not associated with adverse early functional recovery, postoperative complications, or long-term graft and patient survival. These findings were consistent with the primary analysis, despite the original imbalance in group sizes.

## Discussion

In this retrospective cohort study of 251 SPKT recipients, we systematically evaluated the impact of donor SIH on postoperative outcomes. Our findings indicate that grafts from SIH donors exhibited early functional recovery comparable to that of NG donors. No significant differences were observed in key postoperative metabolic parameters-including fasting glucose, C-peptide, HbA1c, and insulin levels-or in renal graft function as measured by serum creatinine. Similarly, complication rates, such as acute rejection, graft thrombosis, and surgical fistulas, did not differ between groups. Most importantly, long-term graft and patient survival, assessed via Kaplan–Meier analysis, were equivalent. Multivariable Cox regression analysis, adjusting for donor age, donor BMI, donor hypertension, cold ischemia time, recipient age, recipient BMI, dialysis duration, and HLA mismatch, confirmed that donor SIH status was not an independent risk factor for graft failure or patient death. Overall, within the constraints of this single-center retrospective analysis, we did not detect evidence of an association between donor SIH and graft viability or clinical success in this SPKT cohort. However, the wide confidence intervals around our effect estimates preclude definitive conclusions.

The clinical significance of these findings is substantial, particularly given the persistent organ shortage. Our study supports the safe expansion of donor selection criteria to include individuals with SIH, thereby increasing the available donor pool without compromising transplant outcomes. The mechanisms underlying the observed lack of association between donor SIH and graft outcomes remain unclear and were not directly investigated in this study. Based on previous literature, it has been hypothesized that stress-induced β-cell dysfunction may be reversible-unlike chronic diabetic damage-due to the transient nature of sympathetic activation, cytokine release, and enhanced hepatic gluconeogenesis (18–20). Some experimental studies have suggested that oxidative stress and inflammatory changes associated with SIH may be mitigated after transplantation, potentially allowing functional recovery of islet cells once the graft is revascularized and placed in a normoglycemic, immunomodulated environment (21, 22). However, it is important to emphasize that our study was not designed to test mechanistic hypotheses directly: we did not measure inflammatory mediators, oxidative stress parameters, or perform dedicated β-cell function tests beyond standard metabolic monitoring, nor did we conduct correlation analyses between donor glycemic profiles and recipient outcomes. Accordingly, the discussion of potential mechanisms is speculative, based on existing literature rather than our own data, and should not be interpreted as providing mechanistic insight into potential mechanisms.

Our findings demonstrate that, in carefully selected donors, SIH is not associated with inferior graft outcomes. Future prospective studies incorporating serial measurements of inflammatory mediators, oxidative stress markers, and dynamic β-cell function tests are needed to clarify the biological pathways involved. Our conclusions align with emerging evidence that challenges traditional caution regarding SIH donors. Previous guidelines have suggested that donor insulin use should not automatically preclude pancreas donation, yet clinical data remain limited and somewhat contradictory (10, 23). Some single-center reports raised theoretical concerns about β-cell exhaustion and postoperative hyperglycemia, whereas larger registry analyses have not identified SIH as a significant predictor of graft failure (24–26). The present study adds detailed metabolic and survival data to this discourse, reinforcing the notion that carefully selected SIH donors-meeting standard criteria for pancreas donation apart from transient glucose elevation-can yield outcomes equivalent to those of normoglycemic donors. Furthermore, our results showed that donor age >45 years as an independent risk factor for pancreas graft failure (HR = 2.31, 95% CI: 1.28-4.17, P = 0.006), whereas donor SIH status was not associated with graft or patient outcomes after adjusting for potential confounders. Although donor age was comparable between the SIH and NG groups at baseline, its emergence as a significant predictor in the overall cohort underscores its importance as a determinant of long-term graft function in SPKT. This finding aligns with broader transplant literature highlighting donor age as a critical factor in donor selection, and suggests that transient metabolic disturbances such as SIH may be less consequential than established donor characteristics (27–29).

This study has a critical limitation in its scope: it provides no direct immunological data. We did not perform flow cytometry for T-cell subsets or regulatory T cells, measure cytokine profiles (e.g., IL-6, TNF-α, IFN-γ), or conduct systematic histopathological scoring of immune cell infiltration (e.g., Banff criteria for rejection) in post-transplant biopsies. The descriptive time-zero biopsy findings (**Supplementary Appendix 1**) are hypothesis-generating only and lack quantification or correlation with clinical outcomes. Consequently, this study cannot answer whether donor SIH affects graft immunogenicity or recipient immune responses. The observed lack of association with clinical outcomes (graft function, rejection rates by clinical criteria, survival) should not be interpreted as evidence of immunological equivalence. Future prospective studies should incorporate serial, protocol-driven immune monitoring-including multiplex cytokine assays, flow cytometric phenotyping of peripheral blood mononuclear cells, and standardized histopathological assessment of protocol biopsies with Banff scoring-to directly test the hypothesis that donor SIH influences post-transplant alloimmunity.

This study has several limitations that deserve consideration. One inherent constraint is the retrospective cohort design, which carries risks of selection bias and unmeasured confounding. Although follow-up data were largely complete with missingness below 3% for any variable, statistical adjustments cannot fully eliminate these biases.

Another limitation concerns the imbalance in group sizes: 210 donors with stress-induced hyperglycemia versus 41 normoglycemic donors. Such imbalance may reduce statistical power and introduce selection bias. To mitigate this, we performed propensity score matching as a sensitivity analysis. After 1:1 matching, all covariates were well balanced, and the matched cohort (82 patients) yielded results consistent with the primary findings. However, this matched sample remains modest in size, limiting power to detect modest differences or uncommon but clinically important events such as graft loss or thrombosis. A post-hoc power calculation showed that the matched cohort had 80% power to detect a hazard ratio of 2.3 for pancreas graft failure, but smaller differences (e.g., HR below 1.8) would require a larger sample. Thus, while the similar rates of thrombosis (5.7% vs. 7.3%) are reassuring, we cannot exclude a small but clinically meaningful increase in risk associated with donor SIH. Larger, prospective studies with balanced groups are needed for confirmation.

The retrospective design also precluded systematic oral glucose tolerance testing, raising the possibility that some donors classified as SIH may have had undiagnosed dysglycemia or impaired glucose tolerance. We defined SIH based on sustained hyperglycemia and insulin requirement-supported by detailed glycemic profiles-rather than a single measurement, yet misclassification cannot be entirely ruled out.

Our primary definition of SIH relied solely on glucose criteria to minimize practice-dependent variability. Insulin initiation thresholds vary across centers and are influenced by factors beyond glucose levels, such as procurement timing and hemodynamic stability. Mandating insulin therapy as an inclusion criterion would have introduced center-specific bias. Nonetheless, the subset of donors who received insulin was inevitably shaped by local ICU protocols, as was the intensity of therapy. The frequency of glucose monitoring and glycemic targets also differ between centers. Consequently, classification by insulin use is not independent of center-level practices, introducing potential bias that affects secondary analyses involving insulin exposure. Reassuringly, sensitivity analyses incorporating insulin therapy (**Supplementary Table 3**) yielded results consistent with the primary analysis, suggesting that the observed lack of association is robust to this aspect of donor management. Residual confounding remains possible.

Follow-up for survival analysis extended up to 9.2 years, but this period may still be insufficient to detect very long-term metabolic sequelae or late graft dysfunction. The cohort was also ethnically homogeneous (over 99% Han Chinese), limiting generalizability to other racial and ethnic populations. Future multi-center studies across diverse geographic regions are needed to validate our conclusions.

Several center-specific practices further constrain external validity. Our median cold ischemia time of 3.4 hours is substantially shorter than the 6-8 hours commonly reported in multi-center registries, reflecting local donor procurement and a dual surgical team strategy to minimize ischemic injury. While this optimizes graft viability, it remains unknown whether the observed lack of association between donor SIH and graft outcomes persists under prolonged cold ischemia. Moreover, our donor selection criteria were relatively strict, excluding donors with body mass index below 18.5 or above 30 kg/m², as well as those with significant hemodynamic instability or prolonged intensive care unit stays. Our donor management protocol included intensive glycemic control with insulin infusion for sustained hyperglycemia, which may differ from less aggressive approaches elsewhere. Our surgical technique-particularly meticulous reconstruction of the splenic artery and vein to preserve pancreatic tail perfusion-represents a single-center practice that may not be uniformly adopted. These center-specific factors underscore the need for multi-center studies with standardized protocols.

The recipient cohort predominantly consisted of patients with type 2 diabetes (approximately 90%). This pattern differs markedly from typical Western SPKT populations and reflects regional differences in diabetes epidemiology. In China, type 2 diabetes accounts for 90-95% of all diabetes cases, leading to a large absolute number of type 2 diabetic patients with end-stage renal disease (15). Our center’s previously published recipient selection criteria further shaped this demographic (17). This feature limits direct generalizability to type 1-predominant cohorts, yet it addresses an important knowledge gap, as outcomes in type 2 diabetic SPKT recipients remain less well characterized globally. The impact of recipient diabetes type on the association between donor SIH and graft outcomes warrants investigation in more diverse cohorts.

The time-zero pancreas biopsies described in **Supplementary Appendix 1** warrant careful contextualization. These biopsies were performed as a research sub-study on a subset of donors and do not represent routine clinical practice. The histopathological and ultrastructural analyses were purely descriptive and hypothesis-generating, without systematic quantification or formal statistical comparisons. Consequently, the observed findings-such as focal mitochondrial changes in a minority of SIH donors-cannot be correlated with clinical outcomes and should not be interpreted as providing mechanistic support for the study’s primary conclusions.

Finally, this study lacks any direct immunological assessment. We did not measure recipient immune cell subsets, cytokine profiles, or donor-specific antibodies. The clinical diagnosis of acute rejection was based on standard criteria (functional changes and/or biopsy when clinically indicated); protocol biopsies or systematic immune monitoring were not performed. Therefore, our findings are confined to clinical endpoints and do not inform on potential differences in graft immunogenicity or the severity and subclinical burden of rejection. Future prospective studies should incorporate protocol-driven immune monitoring to address these questions directly.

## Conclusion

In summary, this was a retrospective, single-center cohort study with substantial limitations in statistical power. Within these constraints, we did not detect evidence that donor SIH is associated with worse early metabolic recovery, higher complication rates, or inferior long-term graft and patient survival following SPKT. These observational data do not establish equivalence. The study had limited power to detect modest but clinically important differences. This is particularly true for rare events such as graft thrombosis. Consequently, clinically meaningful differences cannot be excluded. The findings should be viewed as hypothesis-generating. They must await confirmation in larger, prospective multi-center studies.

## Data Availability

The raw data supporting the conclusions of this article will be made available by the authors, without undue reservation.

## Supplementary material

Supplementary Figure 1 Illustration of splenic artery and vein reconstruction technique.

Supplementary Figure 2 Cumulative incidence function (CIF) curves for graft failure accounting for death as a competing risk. **(A)** CIF curves for kidney graft failure, with death as a competing event. The curves for grafts from donors with stress-induced hyperglycemia (SIH, solid line) and normoglycemic donors (NG, dashed line) are nearly superimposed. **(B)** CIF curves for pancreas graft failure, with death as a competing event.

Supplementary Figure 3 Cumulative incidence functions (CIF) for graft failure accounting for death as a competing risk in the propensity score-matched cohort. **(A)** Kidney graft failure. The cumulative incidence of kidney graft failure over the follow-up period is shown for recipients of grafts from donors with stress-induced hyperglycemia (SIH, solid blue line) versus normoglycemic donors (NG, dashed red line). No significant difference was observed between groups (subdistribution hazard ratio [SHR] = 1.09, 95% CI: 0.49-2.42, P = 0.831). **(B)** Pancreas graft failure. The cumulative incidence of pancreas graft failure was comparable between the SIH and NG groups (SHR = 1.04, 95% CI: 0.46-2.35, P = 0.925). Shaded areas represent 95% confidence bands.

Supplementary Video 1 Surgical procedure for simultaneous pancreas-kidney transplantation (SPKT).

## References

[1] Cao Y Liu X Lan X Ni K Li L Fu Y . Simultaneous pancreas and kidney transplantation for end-stage kidney disease patients with type 2 diabetes mellitus: a systematic review and meta-analysis. Langenbecks Arch Surg. (2022) 407:909–25. doi: 10.1007/s00423-021-02249-y 34279713 PMC9151548

[2] Huber S Fridell JA . Simultaneous pancreas and kidney transplant vs. pancreas after kidney transplantation: is one better? Curr Opin Organ Transplant. (2025) 30:273–8. doi: 10.1097/mot.0000000000001229 40314358

[3] Mauer M Fioretto P . Pancreas transplantation and reversal of diabetic nephropathy lesions. Med Clin North Am. (2013) 97:109–14. doi: 10.1016/j.mcna.2012.10.009 23290733 PMC3646375

[4] Messner F Etra JW Yu Y Massie AB Jackson KR Brandacher G . Outcomes of simultaneous pancreas and kidney transplantation based on donor resuscitation. Am J Transplant. (2020) 20:1720–8. doi: 10.1111/ajt.15808 32026618 PMC12404684

[5] Vedantam D Poman DS Motwani L Asif N Patel A Anne KK . Stress-induced hyperglycemia: consequences and management. Cureus. (2022) 14:e26714. doi: 10.7759/cureus.26714 35959169 PMC9360912

[6] Marvin MR Morton V . Glycemic control and organ transplantation. J Diabetes Sci Technol. (2009) 3:1365–72. doi: 10.1177/193229680900300616 20144390 PMC2787036

[7] Newsholme P Keane KN Carlessi R Cruzat V . Oxidative stress pathways in pancreatic β-cells and insulin-sensitive cells and tissues: importance to cell metabolism, function, and dysfunction. Am J Physiol Cell Physiol. (2019) 317:C420–33. doi: 10.1152/ajpcell.00141.2019 31216193

[8] Vinkers MT Rahmel AO Slot MC Smits JM Schareck WD . How to recognize a suitable pancreas donor: a eurotransplant study of preprocurement factors. Transplant Proc. (2008) 40:1275–8. doi: 10.1016/j.transproceed.2008.03.142 18589086

[9] Axelrod DA Sung RS Meyer KH Wolfe RA Kaufman DB . Systematic evaluation of pancreas allograft quality, outcomes and geographic variation in utilization. Am J Transplant. (2010) 10:837–45. doi: 10.1111/j.1600-6143.2009.02996.x 20121753

[10] Shapey IM Summers A Khambalia H Yiannoullou P Fullwood C Hanley NA . Donor insulin therapy in intensive care predicts early outcomes after pancreas transplantation. Diabetologia. (2021) 64:1375–84. doi: 10.1007/s00125-021-05411-9 33665687 PMC8099796

[11] Muñoz-Bellvís L López-Sánchez J . Donor risk factors in pancreas transplantation. World J Transplant. (2020) 10:372–80. doi: 10.5500/wjt.v10.i12.372 PMC7769731 33437670

[12] Fridell JA Rogers J Stratta RJ . The pancreas allograft donor: current status, controversies, and challenges for the future. Clin Transplant. (2010) 24:433–49. doi: 10.1111/j.1399-0012.2010.01253.x 20384731

[13] Zhang L Chen Z Lai X Ma J Fang J Guo Y . The homolateral simultaneous pancreas-kidney transplantation: a single-center experience in China. Ann Transl Med. (2019) 7:629. doi: 10.21037/atm.2019.10.117 31930030 PMC6944542

[14] Zhang L Li L Zhang Y Yang X Jiang X Yang H . Different biopsy techniques for different pancreas graft locations after homolateral simultaneous pancreas-kidney transplantation. Gland Surg. (2023) 12:324–33. doi: 10.21037/gs-22-414 37057047 PMC10086771

[15] Dong C Wu G Li H Qiao Y Gao S . Type 1 and type 2 diabetes mortality burden: predictions for 2030 based on Bayesian age-period-cohort analysis of China and global mortality burden from 1990 to 2019. J Diabetes Investig. (2024) 15:623–33. doi: 10.1111/jdi.14146 38265170 PMC11060160

[16] Xu Y Wang L He J Bi Y Li M Wang T . Prevalence and control of diabetes in Chinese adults. JAMA. (2013) 310:948–59. doi: 10.1001/jama.2013.168118 24002281

[17] Liu L Xiong Y Zhang T Fang J Zhang L Li G . Effect of simultaneous pancreas-kidney transplantation on blood glucose level for patients with end-stage renal disease with type 1 and type 2 diabetes. Ann Transl Med. (2019) 7:631. doi: 10.21037/atm.2019.10.106 31930032 PMC6944581

[18] Dinić S Arambašić JJ Uskoković A Mihailović M Grdović N Tolić A . Oxidative stress-mediated beta cell death and dysfunction as a target for diabetes management. Front Endocrinol (Lausanne). (2022) 13:1006376. doi: 10.3389/fendo.2022.1006376 36246880 PMC9554708

[19] Berbudi A Rahmadika N Tjahjadi AI Ruslami R . Type 2 diabetes and its impact on the immune system. Curr Diabetes Rev. (2020) 16:442–9. doi: 10.2174/18756417mtaxgodqqy PMC7475801 31657690

[20] Gornik I Vujaklija-Brajkovic A Renar IP Gasparovic V . A prospective observational study of the relationship of critical illness associated hyperglycaemia in medical ICU patients and subsequent development of type 2 diabetes. Crit Care. (2010) 14:R130. doi: 10.1186/cc9101 20615210 PMC2945097

[21] Aljiffry M Hassanain M Schricker T Shaheen M Nouh T Lattermann R . Effect of insulin therapy using hyper-insulinemic normoglycemic clamp on inflammatory response in brain dead organ donors. Exp Clin Endocrinol Diabetes. (2016) 124:318–23. doi: 10.1055/s-0042-101240 27050068

[22] Sally MB Ellis MK Hutchens M Groat T Swanson E Patel MS . Deceased organ donor factors influencing pancreatic graft transplantation and survival. Clin Transplant. (2019) 33:e13571. doi: 10.1111/ctr.13571 31001850 PMC8034871

[23] Ventura-Aguiar P Montagud-Marrahi E Amor AJ Diekmann F . Donor insulin use during stay in the intensive care unit should not preclude pancreas transplantation. Diabetologia. (2021) 64:2122–3. doi: 10.1007/s00125-021-05479-3 34052854

[24] Shapey IM Summers A Yiannoullou P Bannard-Smith J Augustine T Rutter MK . Insulin therapy in organ donation and transplantation. Diabetes Obes Metab. (2019) 21:1521–8. doi: 10.1111/dom.13728 30924574

[25] Shin S Han DJ Kim YH Han S Choi BH Jung JH . Long-term effects of delayed graft function on pancreas graft survival after pancreas transplantation. Transplantation. (2014) 98:1316–22. doi: 10.1097/tp.0000000000000214 24839896

[26] Alfaro VL Junior RM Rangel ÉB Modelli LG Viana LA Cristelli MP . Assessing the influence of graft loss on 4-year patient survival after simultaneous pancreas-kidney transplantation: Kaplan-Meier versus competing risk analysis model. Clin Transplant. (2024) 38:e15298. doi: 10.1111/ctr.15298 38545918

[27] Sharda B Jay CL Gurung K Harriman D Gurram V Farney AC . Improved surgical outcomes following simultaneous pancreas-kidney transplantation in the contemporary era. Clin Transplant. (2022) 36:e14792. doi: 10.1111/ctr.14792 36029250 PMC10078434

[28] Almeida C Sala I Malheiro J Correia S Silvano J Ribeiro C . Impact of donor age on long-term outcomes in simultaneous pancreas-kidney transplantation. Transplant Proc. (2023) 55:1404–7. doi: 10.1016/j.transproceed.2023.04.017 37230903

[29] Dias BF Marques RC Cardoso C Faria V Domingues P Ribeiro C . Surgical complications and technical failure of simultaneous pancreas-kidney transplantation: a 22-year experience from a single center. Clin Transplant. (2024) 38:e15339. doi: 10.1111/ctr.15339 38775413

