# Correction: Impact of donor stress-induced hyperglycemia on early graft outcomes in simultaneous pancreas-kidney transplantation: a retrospective cohort study

**DOI:** 10.3389/fimmu.2026.1918588

**Published:** 2026-07-06

**Authors:** Luhao Liu, Rongxin Chen, Yuhe Guo, Ziqing Guo, Jiao Wan, Guanghui Li, Jiali Fang, Junjie Ma, Zheng Chen

**Affiliations:** 1 Department of Organ Transplantation, The Second Affiliated Hospital, Guangzhou Medical University, Guangzhou, China; 2 Guangzhou University, Guangzhou, China; 3 General Surgery Department, Wangniudun Hospital, Dongguan, China

**Keywords:** donor selection, graft survival, organ donor, simultaneous pancreas-kidney transplantation, stress hyperglycemia

Author “Luhao Liu, Rongxin Chen, Yuhe Guo” was erroneously omitted as equal contributing author.

“^†^Luhao Liu, Rongxin Chen, and Yuhe Guo contributed equally to this work”.

The original version of this article has been updated.

