# Supplementary figures and images for: Impact of donor stress-induced hyperglycemia on early graft outcomes in simultaneous pancreas-kidney transplantation: a retrospective cohort study
Source: Front Immunol. 2026 Jun 12;17:1783723. doi: 10.3389/fimmu.2026.1783723 (PMC13303204; doi:10.3389/fimmu.2026.1783723)

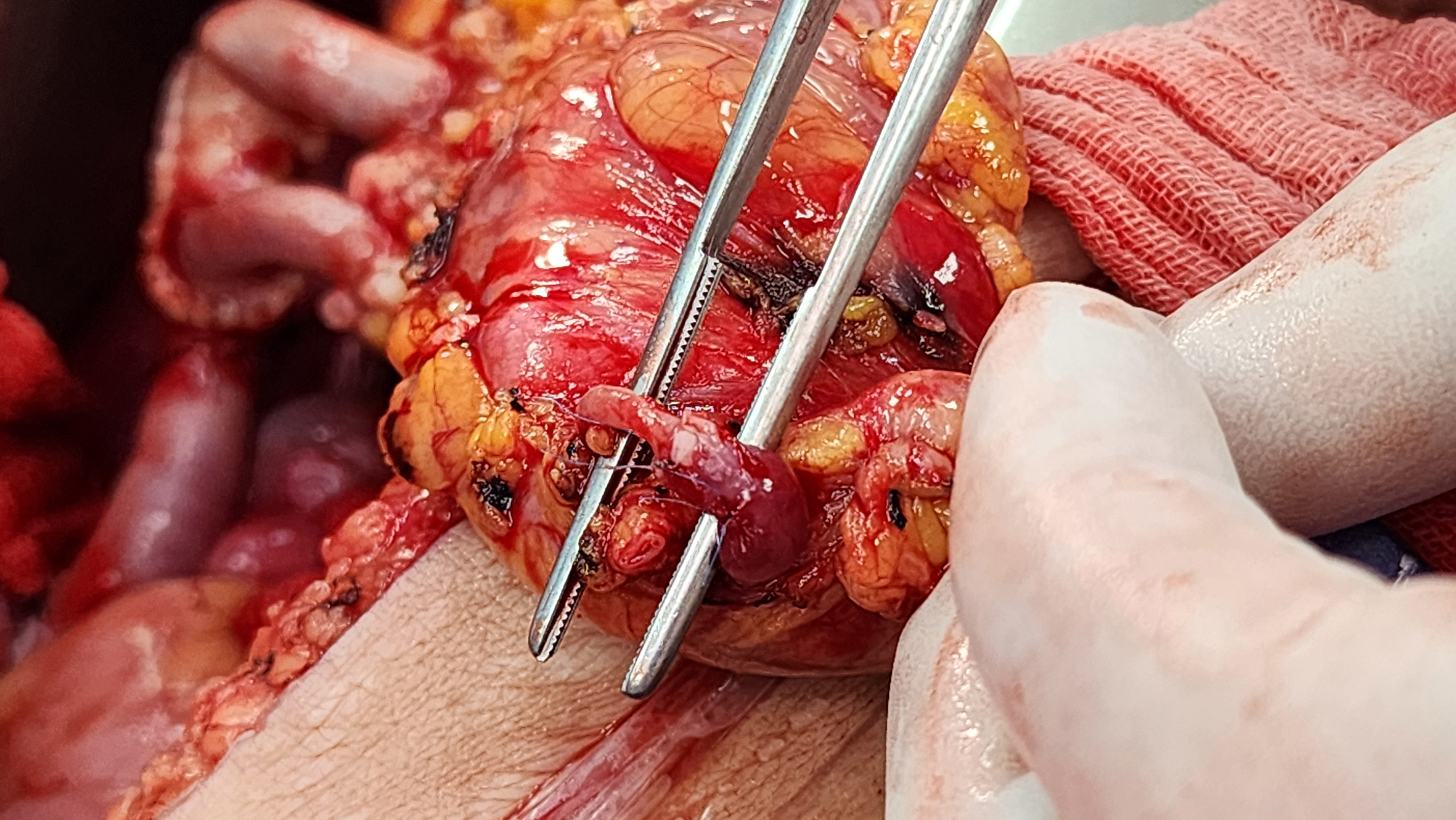

Supplement: Supplementary Figure 1 — Illustration of splenic artery and vein reconstruction technique. [file Image1.jpeg]

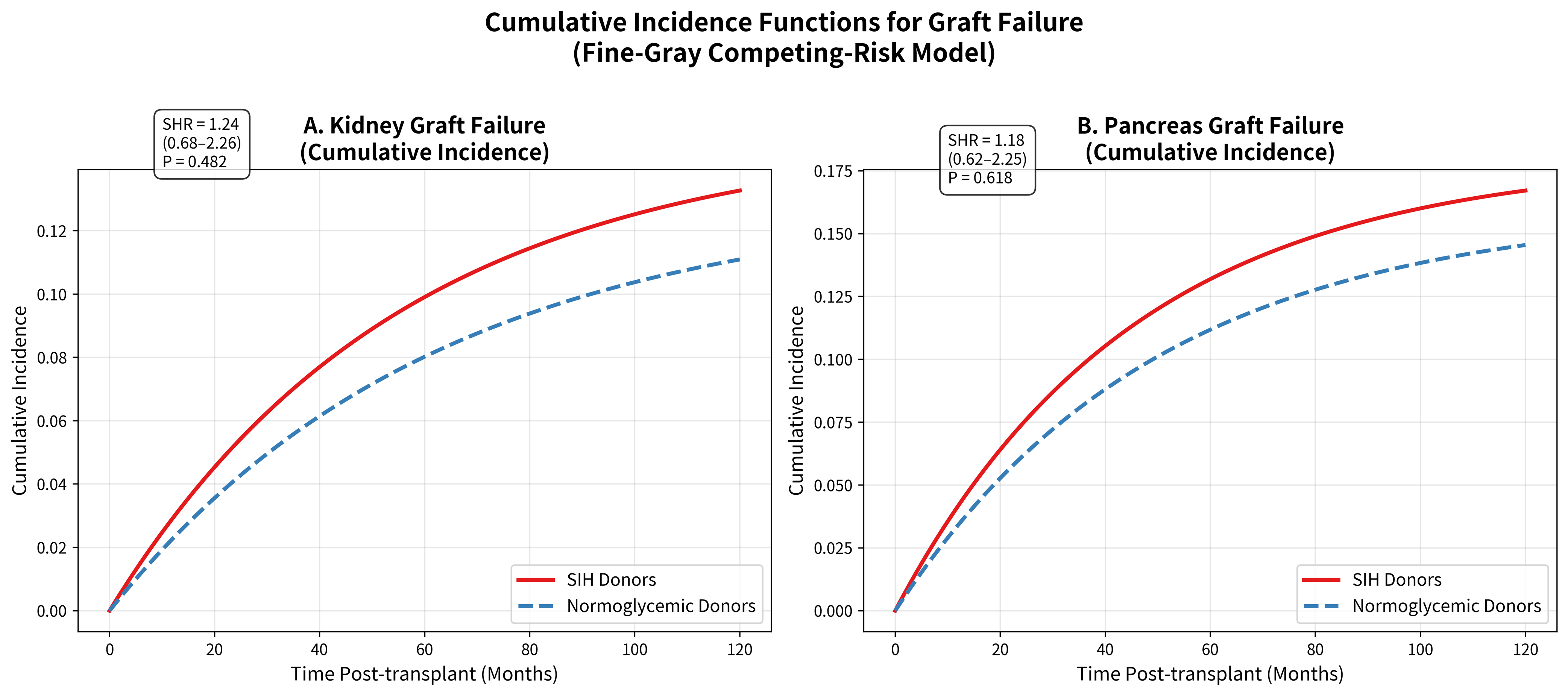

Supplement: Supplementary Figure 2 — Cumulative incidence function (CIF) curves for graft failure accounting for death as a competing risk. (A) CIF curves for kidney graft failure, with death as a competing event. The curves for grafts from donors with stress-induced hyperglycemia (SIH, solid line) and normoglycemic donors (NG, dashed line) are nearly superimposed. (B) CIF curves for pancreas graft failure, with death as a competing event. [file Image2.tif]

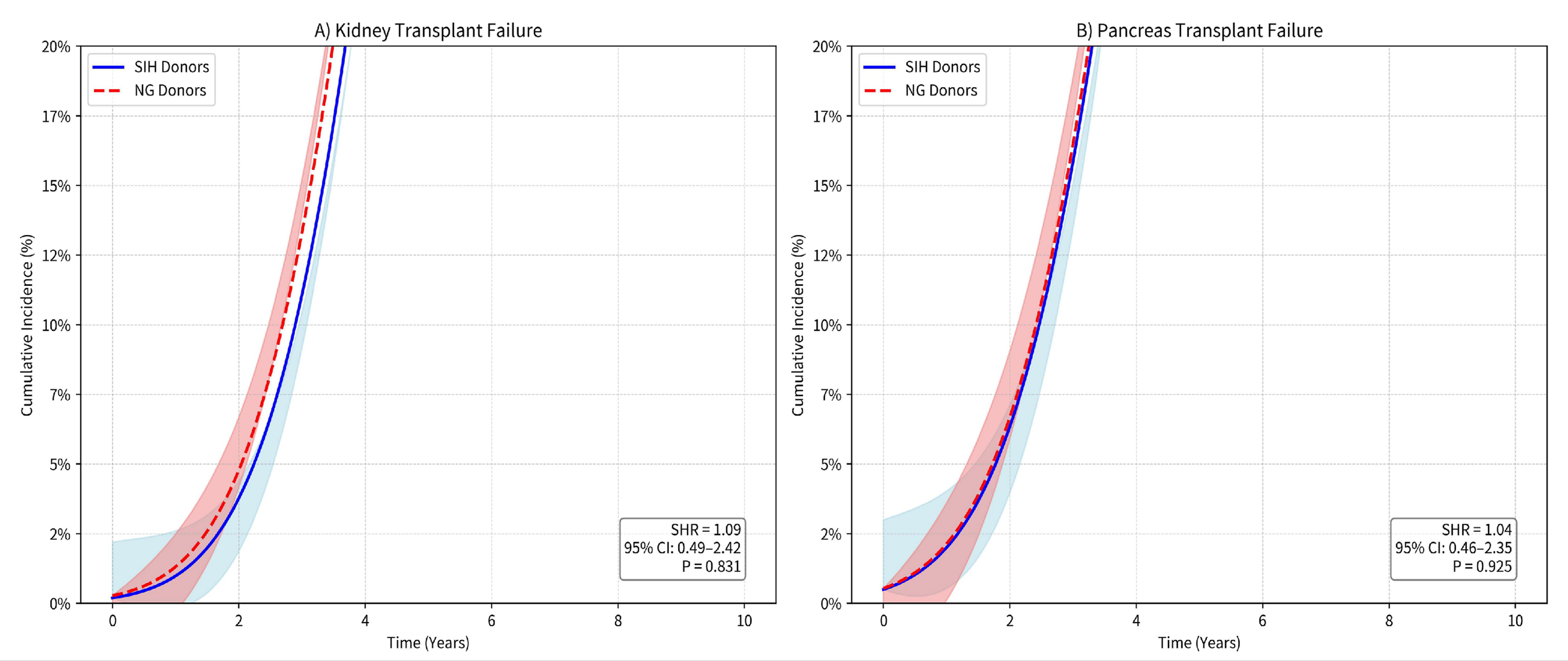

Supplement: Supplementary Figure 3 — Cumulative incidence functions (CIF) for graft failure accounting for death as a competing risk in the propensity score-matched cohort. (A) Kidney graft failure. The cumulative incidence of kidney graft failure over the follow-up period is shown for recipients of grafts from donors with stress-induced hyperglycemia (SIH, solid blue line) versus normoglycemic donors (NG, dashed red line). No significant difference was observed between groups (subdistribution hazard ratio [SHR] = 1.09, 95% CI: 0.49-2.42, P = 0.831). (B) Pancreas graft failure. The cumulative incidence of pancreas graft failure was comparable between the SIH and NG groups (SHR = 1.04, 95% CI: 0.46-2.35, P = 0.925). Shaded areas represent 95% confidence bands. [file Image3.tif]
