# Supplementary material for: Impact of donor stress-induced hyperglycemia on early graft outcomes in simultaneous pancreas-kidney transplantation: a retrospective cohort study
Source: Front Immunol. 2026 Jun 12;17:1783723. doi: 10.3389/fimmu.2026.1783723 (PMC13303204; doi:10.3389/fimmu.2026.1783723)
Supplement: Supplementary file 5 [file Table1.doc]

****Supplementary Table 1.**** presents the comparison of these additional donor characteristics between the SIH and NG groups.

| Variable | SIH Group (n=210) | NG Group (n=41) | P value |
| --- | --- | --- | --- |
| ****Serum amylase (U/L)****, median (IQR) | 68 (45-112) | 62 (41-98) | 0.284 |
| ****Serum lipase (U/L)****, median (IQR) | 42 (28-76) | 38 (24-65) | 0.312 |
| ****Insulin requirements (units/24h)****, median (IQR) | 42 (18-76) | 0 (0-0) | <0.001 |
| ****Vasopressor support****, n (%) | 178 (84.8%) | 32 (78.0%) | 0.281 |
| - Single vasopressor | 102 (48.6%) | 21 (51.2%) |  |
| - Multiple vasopressors | 76 (36.2%) | 11 (26.8%) |  |
| ****Hemodynamic instability****, n (%) | 92 (43.8%) | 16 (39.0%) | 0.568 |
| ****ICU length of stay (days)****, median (IQR) | 4 (2-7) | 4 (2-6) | 0.672 |
