# Supplementary material for: Impact of donor stress-induced hyperglycemia on early graft outcomes in simultaneous pancreas-kidney transplantation: a retrospective cohort study
Source: Front Immunol. 2026 Jun 12;17:1783723. doi: 10.3389/fimmu.2026.1783723 (PMC13303204; doi:10.3389/fimmu.2026.1783723)
Supplement: Supplementary file 6 [file Table2.doc]

Supplementary Table 2. Univariable analysis of additional donor variables for pancreas graft failure.

| Variable | HR (95% CI) | P value |
| --- | --- | --- |
| Donor amylase (per 10 U/L increase) | 1.02 (0.98–1.06) | 0.342 |
| Donor lipase (per 10 U/L increase) | 1.03 (0.97–1.09) | 0.287 |
| Insulin requirements (per 10 units/24h) | 1.01 (0.96–1.06) | 0.712 |
| Vasopressor support (any vs. none) | 1.24 (0.68–2.26) | 0.482 |
| Multiple vasopressors (vs. none) | 1.38 (0.71–2.68) | 0.341 |
| Hemodynamic instability (yes vs. no) | 1.18 (0.69–2.02) | 0.548 |
| ICU length of stay (per day) | 1.03 (0.95–1.12) | 0.452 |
