# Supplementary material for: Impact of donor stress-induced hyperglycemia on early graft outcomes in simultaneous pancreas-kidney transplantation: a retrospective cohort study
Source: Front Immunol. 2026 Jun 12;17:1783723. doi: 10.3389/fimmu.2026.1783723 (PMC13303204; doi:10.3389/fimmu.2026.1783723)
Supplement: Supplementary file 7 [file Table3.doc]

### Supplementary Table 3. Sensitivity Analysis Using Definition A (Peak Glucose >11.1 mmol/L AND Insulin Therapy)

| Outcome | SIH Group (Def A) | NG Group | P value |
| --- | --- | --- | --- |
| ****Longitudinal graft function**** |  |  |  |
| - Fasting glucose (group effect) | - | - | 0.482 |
| - HbA1c (group effect) | - | - | 0.631 |
| - C-peptide (group effect) | - | - | 0.557 |
| - Serum creatinine (group effect) | - | - | 0.702 |
| ****Postoperative complications**** |  |  |  |
| - Delayed graft function, n (%) | 11 (5.9%) | 3 (7.3%) | 0.724 |
| - Kidney rejection, n (%) | 15 (8.1%) | 3 (7.3%) | 0.861 |
| - Pancreas rejection, n (%) | 14 (7.6%) | 2 (4.9%) | 0.539 |
| - Pancreatic graft thrombosis, n (%) | 10 (5.4%) | 3 (7.3%) | 0.705 |
| ****Graft survival**** |  |  |  |
| - Death-censored kidney graft survival (HR, 95% CI) | 1.18 (0.62–2.24) | Reference | 0.612 |
| - Death-censored pancreas graft survival (HR, 95% CI) | 1.21 (0.58–2.52) | Reference | 0.608 |
| ****Competing risk analysis**** |  |  |  |
| - Kidney graft failure (SHR, 95% CI) | 1.16 (0.60–2.24) | Reference | 0.658 |
| - Pancreas graft failure (SHR, 95% CI) | 1.19 (0.56–2.53) | Reference | 0.646 |

Definition A: SIH = peak glucose >11.1 mmol/L during ICU stay AND receipt of insulin therapy (n=185). NG group unchanged (n=41). Donors originally classified as SIH who did not receive insulin therapy (n=25) were excluded from this analysis.

Abbreviations: SIH, stress-induced hyperglycemia; NG, normoglycemia; HR, hazard ratio; SHR, subdistribution hazard ratio; CI, confidence interval.

Note: Results were consistent with the primary analysis, demonstrating no association between donor SIH (defined by peak glucose >11.1 mmol/L plus insulin therapy) and adverse graft outcomes
