# Supplementary material for: Impact of donor stress-induced hyperglycemia on early graft outcomes in simultaneous pancreas-kidney transplantation: a retrospective cohort study
Source: Front Immunol. 2026 Jun 12;17:1783723. doi: 10.3389/fimmu.2026.1783723 (PMC13303204; doi:10.3389/fimmu.2026.1783723)
Supplement: Supplementary file 8 [file Table4.doc]

### Supplementary Table 4. Sensitivity Analysis Using Definition B (Peak Glucose >11.1 mmol/L Regardless of Insulin Therapy)

| Outcome | SIH Group  (Def B) | NG Group | P value |
| --- | --- | --- | --- |
| ****Longitudinal graft function**** |  |  |  |
| - Fasting glucose (group effect) | - | - | 0.513 |
| - HbA1c (group effect) | - | - | 0.594 |
| - C-peptide (group effect) | - | - | 0.612 |
| - Serum creatinine (group effect) | - | - | 0.678 |
| ****Postoperative complications**** |  |  |  |
| - Delayed graft function, n (%) | 12 (6.1%) | 3 (7.3%) | 0.726 |
| - Kidney rejection, n (%) | 17 (8.6%) | 3 (7.3%) | 0.783 |
| - Pancreas rejection, n (%) | 15 (7.6%) | 2 (4.9%) | 0.542 |
| - Pancreatic graft thrombosis, n (%) | 11 (5.6%) | 3 (7.3%) | 0.711 |
| ****Graft survival**** |  |  |  |
| - Death-censored kidney graft survival (HR, 95% CI) | 1.21 (0.65–2.25) | Reference | 0.548 |
| - Death-censored pancreas graft survival (HR, 95% CI) | 1.16 (0.57–2.36) | Reference | 0.684 |
| ****Competing risk analysis**** |  |  |  |
| - Kidney graft failure (SHR, 95% CI) | 1.19 (0.63–2.25) | Reference | 0.587 |
| - Pancreas graft failure (SHR, 95% CI) | 1.14 (0.55–2.36) | Reference | 0.722 |

****Definition B:**** SIH = peak glucose >11.1 mmol/L during ICU stay, regardless of insulin therapy (n=198). NG group unchanged (n=41). Donors originally classified as SIH with peak glucose ≤11.1 mmol/L but who received insulin for other indications (n=12) were excluded from this analysis.

Abbreviations: SIH, stress-induced hyperglycemia; NG, normoglycemia; HR, hazard ratio; SHR, subdistribution hazard ratio; CI, confidence interval.
Note: Results using the glucose-only definition were consistent with the primary analysis, confirming the robustness of our findings.
