# Supplementary material for: Impact of donor stress-induced hyperglycemia on early graft outcomes in simultaneous pancreas-kidney transplantation: a retrospective cohort study
Source: Front Immunol. 2026 Jun 12;17:1783723. doi: 10.3389/fimmu.2026.1783723 (PMC13303204; doi:10.3389/fimmu.2026.1783723)
Supplement: Supplementary file 9 [file Table5.doc]

### Supplementary Table 5. Sensitivity Analysis Using Definition C (Persistent Hyperglycemia: ≥3 Measurements >11.1 mmol/L Over ≥24 Hours).

| Outcome | SIH Group  (Def C) | NG Group | P value |
| --- | --- | --- | --- |
| ****Longitudinal graft function**** |  |  |  |
| - Fasting glucose (group effect) | - | - | 0.601 |
| - HbA1c (group effect) | - | - | 0.572 |
| - C-peptide (group effect) | - | - | 0.634 |
| - Serum creatinine (group effect) | - | - | 0.715 |
| ****Postoperative complications**** |  |  |  |
| - Delayed graft function, n (%) | 10 (6.2%) | 3 (7.3%) | 0.728 |
| - Kidney rejection, n (%) | 14 (8.6%) | 3 (7.3%) | 0.779 |
| - Pancreas rejection, n (%) | 12 (7.4%) | 2 (4.9%) | 0.738 |
| - Pancreatic graft thrombosis, n (%) | 9 (5.6%) | 3 (7.3%) | 0.708 |
| ****Graft survival**** |  |  |  |
| - Death-censored kidney graft survival (HR, 95% CI) | 1.14 (0.58–2.24) | Reference | 0.702 |
| - Death-censored pancreas graft survival (HR, 95% CI) | 1.09 (0.51–2.33) | Reference | 0.824 |
| ****Competing risk analysis**** |  |  |  |
| - Kidney graft failure (SHR, 95% CI) | 1.12 (0.56–2.24) | Reference | 0.748 |
| - Pancreas graft failure (SHR, 95% CI) | 1.06 (0.49–2.29) | Reference | 0.881 |

****Definition C:**** SIH = at least three glucose measurements >11.1 mmol/L over a minimum of 24 hours during ICU stay (n=162). NG group unchanged (n=41). Donors originally classified as SIH with fewer than three hyperglycemic measurements or duration <24 hours (n=48) were excluded from this analysis.

Abbreviations: SIH, stress-induced hyperglycemia; NG, normoglycemia; HR, hazard ratio; SHR, subdistribution hazard ratio; CI, confidence interval.
Note: Results using the strictest definition of persistent hyperglycemia were consistent with the primary analysis, further supporting the robustness of our conclusions.
