# Supplementary material for: Impact of donor stress-induced hyperglycemia on early graft outcomes in simultaneous pancreas-kidney transplantation: a retrospective cohort study
Source: Front Immunol. 2026 Jun 12;17:1783723. doi: 10.3389/fimmu.2026.1783723 (PMC13303204; doi:10.3389/fimmu.2026.1783723)
Supplement: Supplementary file 10 [file Table6.doc]

Supplementary Table 6. Covariate Balance Before and After Propensity Score Matching.

| Covariate | Before Matching | | | After Matching | | |
| --- | --- | --- | --- | --- | --- | --- |
|  | SIH Group  (n=210) | NG Group  (n=41) | SMD | SIH Group (n=41) | NG Group (n=41) | SMD |
| Donor age (years), mean ± SD | 34.68 ± 12.25 | 32.68 ± 10.87 | 0.172 | 33.42 ± 11.86 | 32.68 ± 10.87 | 0.065 |
| Donor BMI (kg/m²), mean ± SD | 22.80 ± 3.48 | 22.39 ± 3.24 | 0.122 | 22.51 ± 3.31 | 22.39 ± 3.24 | 0.037 |
| Donor hypertension, n (%) | 36 (17.1%) | 5 (12.2%) | 0.139 | 5 (12.2%) | 5 (12.2%) | 0.000 |
| Cause of brain death, n (%) |  |  | 0.084 |  |  | 0.048 |
| - Traumatic brain injury | 124 (59.0%) | 24 (58.5%) |  | 24 (58.5%) | 24 (58.5%) |  |
| - Cerebral hemorrhage | 68 (32.4%) | 14 (34.2%) |  | 14 (34.2%) | 14 (34.2%) |  |
| - Other causes | 18 (8.6%) | 3 (7.3%) |  | 3 (7.3%) | 3 (7.3%) |  |
| Cold ischemia time (h), mean ± SD | 3.45 ± 1.92 | 3.28 ± 1.49 | 0.099 | 3.31 ± 1.58 | 3.28 ± 1.49 | 0.020 |
| Recipient age (years), mean ± SD | 49.40 ± 9.60 | 50.00 ± 11.10 | 0.058 | 49.80 ± 10.20 | 50.00 ± 11.10 | 0.019 |
| Recipient BMI (kg/m²), mean ± SD | 23.70 ± 3.10 | 23.80 ± 2.90 | 0.033 | 23.75 ± 3.05 | 23.80 ± 2.90 | 0.017 |
| Dialysis duration (months), mean ± SD | 14.60 ± 16.80 | 12.10 ± 12.40 | 0.168 | 12.80 ± 13.50 | 12.10 ± 12.40 | 0.054 |
| HLA mismatch, mean ± SD | 2.81 ± 0.86 | 2.71 ± 0.90 | 0.114 | 2.73 ± 0.88 | 2.71 ± 0.90 | 0.023 |

Abbreviations: SIH, stress-induced hyperglycemia; NG, normoglycemia; SD, standard deviation; SMD, standardized mean difference; BMI, body mass index; HLA, human leukocyte antigen.
Note: Standardized mean difference (SMD) < 0.1 indicates good covariate balance after matching.
