# Supplementary material for: Impact of donor stress-induced hyperglycemia on early graft outcomes in simultaneous pancreas-kidney transplantation: a retrospective cohort study
Source: Front Immunol. 2026 Jun 12;17:1783723. doi: 10.3389/fimmu.2026.1783723 (PMC13303204; doi:10.3389/fimmu.2026.1783723)
Supplement: Supplementary file 11 [file Table7.doc]

### Supplementary Table 7. Baseline Characteristics of the Propensity Score-Matched Cohort.

| Characteristic | SIH Group (n=41) | NG Group (n=41) | P value |
| --- | --- | --- | --- |
| ****Donor characteristics**** |  |  |  |
| Age (years), mean ± SD | 33.42 ± 11.86 | 32.68 ± 10.87 | 0.768 |
| BMI (kg/m²), mean ± SD | 22.51 ± 3.31 | 22.39 ± 3.24 | 0.867 |
| Gender (male), n (%) | 24 (58.5%) | 26 (63.4%) | 0.651 |
| Admission blood glucose (mmol/L), median (IQR) | 9.18 (6.92–12.35) | 6.23 (4.78–8.08) | <0.001 |
| Pre-procurement serum creatinine (μmol/L), mean ± SD | 141.6 ± 102.3 | 120.3 ± 88.4 | 0.312 |
| Cause of brain death, n (%) |  |  | 1.000 |
| - Traumatic brain injury | 24 (58.5%) | 24 (58.5%) |  |
| - Cerebral hemorrhage | 14 (34.2%) | 14 (34.2%) |  |
| - Other causes | 3 (7.3%) | 3 (7.3%) |  |
| Hypertension, n (%) | 5 (12.2%) | 5 (12.2%) | 1.000 |
| Warm ischemia time (min), mean ± SD | 1.00 ± 1.08 | 1.02 ± 1.48 | 0.945 |
| Cold ischemia time (h), mean ± SD | 3.31 ± 1.58 | 3.28 ± 1.49 | 0.929 |
| Transplant surgery time (h), mean ± SD | 6.20 ± 0.31 | 6.19 ± 0.29 | 0.879 |
| ****Recipient characteristics**** |  |  |  |
| Age (years), mean ± SD | 49.80 ± 10.20 | 50.00 ± 11.10 | 0.932 |
| BMI (kg/m²), mean ± SD | 23.75 ± 3.05 | 23.80 ± 2.90 | 0.939 |
| Gender (male), n (%) | 35 (85.4%) | 36 (87.8%) | 0.745 |
| Dialysis duration (months), mean ± SD | 12.80 ± 13.50 | 12.10 ± 12.40 | 0.807 |
| Primary disease, n (%) |  |  | 0.763 |
| - Diabetic nephropathy | 34 (82.9%) | 35 (85.4%) |  |
| - Chronic glomerulonephritis | 7 (17.1%) | 6 (14.6%) |  |
| Hypertension, n (%) | 38 (92.7%) | 38 (92.7%) | 1.000 |
| Types of diabetes, n (%) |  |  | 0.723 |
| - Type 1 | 4 (9.8%) | 4 (9.8%) |  |
| - Type 2 | 37 (90.2%) | 37 (90.2%) |  |
| PRA positive, n (%) | 4 (9.8%) | 4 (9.8%) | 1.000 |
| HLA mismatch, mean ± SD | 2.73 ± 0.88 | 2.71 ± 0.90 | 0.919 |
| Dialysis type, n (%) |  |  | 0.871 |
| - No dialysis | 2 (4.9%) | 2 (4.9%) |  |
| - Peritoneal dialysis | 1 (2.4%) | 1 (2.4%) |  |
| - Hemodialysis | 38 (92.7%) | 38 (92.7%) |  |

Abbreviations: SIH, stress-induced hyperglycemia; NG, normoglycemia; SD, standard deviation; IQR, interquartile range; BMI, body mass index; PRA, panel-reactive antibody; HLA, human leukocyte antigen.
