# Supplementary material for: Impact of donor stress-induced hyperglycemia on early graft outcomes in simultaneous pancreas-kidney transplantation: a retrospective cohort study
Source: Front Immunol. 2026 Jun 12;17:1783723. doi: 10.3389/fimmu.2026.1783723 (PMC13303204; doi:10.3389/fimmu.2026.1783723)
Supplement: Supplementary file 12 [file Table8.doc]

### Supplementary Table 8. Longitudinal Analysis of Graft Function in the Propensity Score-Matched Cohort: Model-Estimated Means (95% CI).

| Outcome | Time Point | SIH Group (n=41) | NG Group (n=41) | P value (Group) | P value (Interaction) |
| --- | --- | --- | --- | --- | --- |
| ****Fasting Glucose (mmol/L)**** |  |  |  | 0.512 | 0.428 |
|  | Postop day 7 | 7.58 (7.02–8.14) | 7.51 (6.89–8.13) |  |  |
|  | 1 month | 6.08 (5.64–6.52) | 6.19 (5.67–6.71) |  |  |
|  | 6 months | 5.41 (5.05–5.77) | 5.64 (5.22–6.06) |  |  |
|  | 1 year | 5.48 (5.12–5.84) | 5.81 (5.39–6.23) |  |  |
| ****HbA1c (%)**** |  |  |  | 0.648 | 0.591 |
|  | Postop day 7 | 6.51 (6.23–6.79) | 6.48 (6.14–6.82) |  |  |
|  | 1 month | 6.11 (5.87–6.35) | 6.12 (5.84–6.40) |  |  |
|  | 6 months | 5.69 (5.49–5.89) | 5.73 (5.51–5.95) |  |  |
|  | 1 year | 5.68 (5.48–5.88) | 5.75 (5.53–5.97) |  |  |
| ****Fasting C-peptide (ng/mL)**** |  |  |  | 0.573 | 0.634 |
|  | Postop day 7 | 9.42 (8.43–10.41) | 9.12 (7.93–10.31) |  |  |
|  | 1 month | 7.15 (6.38–7.92) | 6.91 (6.01–7.81) |  |  |
|  | 6 months | 5.03 (4.43–5.63) | 4.95 (4.27–5.63) |  |  |
|  | 1 year | 4.71 (4.18–5.24) | 4.86 (4.27–5.45) |  |  |
| ****Fasting Insulin (μIU/mL)**** |  |  |  | 0.386 | 0.312 |
|  | Postop day 7 | 23.42 (20.18–26.66) | 28.64 (24.86–32.42) |  |  |
|  | 1 month | 18.89 (16.62–21.16) | 18.59 (15.80–21.38) |  |  |
|  | 6 months | 15.98 (14.21–17.75) | 16.24 (14.15–18.33) |  |  |
|  | 1 year | 15.71 (13.98–17.44) | 17.28 (15.23–19.33) |  |  |
| ****Serum Amylase (U/L)**** |  |  |  | 0.612 | 0.503 |
|  | Postop day 7 | 198.6 (168.4–228.8) | 214.9 (175.8–254.0) |  |  |
|  | 1 month | 155.2 (134.8–175.6) | 142.8 (118.1–167.5) |  |  |
|  | 6 months | 106.8 (92.4–121.2) | 106.2 (89.7–122.7) |  |  |
|  | 1 year | 92.5 (80.8–104.2) | 100.5 (86.4–114.6) |  |  |
| ****Serum Creatinine (μmol/L)**** |  |  |  | 0.683 | 0.745 |
|  | Preoperative | 835.6 (780.2–891.0) | 841.7 (773.2–910.2) |  |  |
|  | Postop day 7 | 185.3 (158.7–211.9) | 168.4 (135.7–201.1) |  |  |
|  | 1 month | 135.2 (122.4–148.0) | 129.5 (114.0–145.0) |  |  |
|  | 6 months | 132.1 (120.5–143.7) | 130.8 (117.5–144.1) |  |  |
|  | 1 year | 135.4 (124.2–146.6) | 135.9 (122.8–149.0) |  |  |

Abbreviations: SIH, stress-induced hyperglycemia; NG, normoglycemia; CI, confidence interval; HbA1c, hemoglobin A1c.
Note: Data are presented as model-estimated means (95% CI) from linear mixed models for repeated measures. All P values for group effects and group-by-time interactions were non-significant (P > 0.05).
