# Supplementary material for: Impact of donor stress-induced hyperglycemia on early graft outcomes in simultaneous pancreas-kidney transplantation: a retrospective cohort study
Source: Front Immunol. 2026 Jun 12;17:1783723. doi: 10.3389/fimmu.2026.1783723 (PMC13303204; doi:10.3389/fimmu.2026.1783723)
Supplement: Supplementary file 13 [file Table9.doc]

### Supplementary Table 9. Postoperative Complications in the Propensity Score-Matched Cohort.

| Complication | SIH Group (n=41) | NG Group (n=41) | P value |
| --- | --- | --- | --- |
| DGF | 3 (7.3%) | 3 (7.3%) | 1.000 |
| Kidney rejection | 3 (7.3%) | 3 (7.3%) | 1.000 |
| Pancreas rejection | 2 (4.9%) | 2 (4.9%) | 1.000 |
| Enteric fistula | 1 (2.4%) | 2 (4.9%) | 0.556 |
| Pancreatic fistula | 1 (2.4%) | 1 (2.4%) | 1.000 |
| Pancreatic graft thrombosis | 2 (4.9%) | 3 (7.3%) | 0.644 |
| Intestinal tract bleeding | 5 (12.2%) | 6 (14.6%) | 0.745 |
| Intestinal obstruction | 2 (4.9%) | 3 (7.3%) | 0.644 |

Abbreviations: SIH, stress-induced hyperglycemia; NG, normoglycemia; DGF, delayed graft function.
Note: Data are presented as n (%). No significant differences were observed between groups for any complication.
