# Supplementary material for: Impact of donor stress-induced hyperglycemia on early graft outcomes in simultaneous pancreas-kidney transplantation: a retrospective cohort study
Source: Front Immunol. 2026 Jun 12;17:1783723. doi: 10.3389/fimmu.2026.1783723 (PMC13303204; doi:10.3389/fimmu.2026.1783723)
Supplement: Supplementary file 14 [file Table10.doc]

****Supplementary Table 10. Multivariable Cox Regression Analysis for Graft Failure and Patient Death****

| Covariate | Death-Censored Kidney Graft Failure | | Death-Censored Pancreas Graft Failure | | Patient Death | |
| --- | --- | --- | --- | --- | --- | --- |
|  | HR (95% CI) | P value | HR (95% CI) | P value | HR (95% CI) | P value |
| Donor SIH status (yes vs. no) | 1.24 (0.68–2.26) | 0.482 | 1.18 (0.62–2.25) | 0.618 | 0.92 (0.45–1.88) | 0.821 |
| Donor age >45 years | 1.52 (0.92–2.51) | 0.102 | 2.31 (1.28–4.17) | 0.006 | 1.18 (0.64–2.18) | 0.601 |
| Donor BMI (per 1 kg/m²) | 1.02 (0.95–1.09) | 0.582 | 1.03 (0.96–1.11) | 0.412 | 0.98 (0.90–1.07) | 0.672 |
| Donor hypertension (yes vs. no) | 1.18 (0.64–2.18) | 0.598 | 1.22 (0.62–2.40) | 0.564 | 1.31 (0.68–2.52) | 0.421 |
| Cold ischemia time (per hour) | 1.04 (0.92–1.18) | 0.512 | 1.06 (0.93–1.21) | 0.382 | 1.02 (0.88–1.18) | 0.798 |
| Recipient age (per year) | 1.01 (0.98–1.04) | 0.542 | 1.00 (0.97–1.03) | 0.892 | 1.03 (0.99–1.07) | 0.112 |
| Recipient BMI (per 1 kg/m²) | 0.98 (0.92–1.05) | 0.582 | 0.97 (0.90–1.05) | 0.462 | 0.99 (0.92–1.07) | 0.812 |
| Dialysis duration (per month) | 1.00 (0.99–1.01) | 0.682 | 1.00 (0.99–1.01) | 0.712 | 1.01 (0.99–1.02) | 0.322 |
| HLA mismatch (per 1 mismatch) | 1.12 (0.88–1.43) | 0.352 | 1.08 (0.84–1.39) | 0.552 | 1.05 (0.79–1.40) | 0.742 |

Abbreviations: SIH, stress-induced hyperglycemia; BMI, body mass index; HLA, human leukocyte antigen; HR, hazard ratio; CI, confidence interval.
