# Supplementary material for: Impact of donor stress-induced hyperglycemia on early graft outcomes in simultaneous pancreas-kidney transplantation: a retrospective cohort study
Source: Front Immunol. 2026 Jun 12;17:1783723. doi: 10.3389/fimmu.2026.1783723 (PMC13303204; doi:10.3389/fimmu.2026.1783723)

**Supplementary Appendix 1. Exploratory Histopathological Observations from Time-Zero Pancreas Biopsies**

**Methods**

As an exploratory sub-study, time-zero pancreas graft biopsies were obtained immediately after procurement from a subset of donors prior to implantation. In our center, time-zero pancreas biopsies are not performed as routine clinical practice. These biopsies were obtained as part of a research sub-study under an approved institutional ethics protocol.

Indications and selection criteria: Biopsies were performed on a subset of donors based on the following considerations:

Donor SIH status: Donors with SIH were preferentially included to assess potential morphological differences compared with normoglycemic donors.

Mildly elevated amylase/lipase: Donors with borderline elevated pancreatic enzymes were considered, as these may indicate subclinical pancreatic injury that could benefit from histopathological assessment.

Assessment of β-cell reserve: In donors with SIH, we aimed to evaluate whether transient hyperglycemia was associated with any detectable morphological changes in islet cells.

Tissue availability: Biopsies were performed on consecutive donors during the study period for whom adequate tissue could be procured for research purposes without interfering with clinical transplantation.

Consistency: Light microscopy was performed on 87 donors (69 SIH, 18 NG); transmission electron microscopy was performed on a subset of 24 donors (18 SIH, 6 NG) with adequate sample quality. Findings are presented descriptively; no formal quantification or statistical comparisons were conducted, given the exploratory nature of this analysis.

**Results**

Light Microscopy

Hematoxylin and eosin staining demonstrated preserved pancreatic histoarchitecture in both SIH and NG donors. Acinar cells contained abundant zymogen granules, and islets of Langerhans appeared morphologically normal. No qualitative differences were observed between groups.

Electron Microscopy

In the majority of samples from both groups, subcellular organelles-including zymogen granules, endoplasmic reticulum, mitochondria, and nuclei-appeared similar. However, in a minority of cells from 5 of the 18 SIH donors (28%), focal mitochondrial alterations were observed, characterized by mild swelling and focal disruption of cristae. These changes were not widespread and were not accompanied by other ultrastructural abnormalities. No such changes were observed in any of the 6 NG donors.

Discussion

The findings presented here are purely descriptive and hypothesis-generating. We agree that the observed mitochondrial changes are described only qualitatively and that no formal correlation analyses were performed.

Exploratory nature: This analysis was intended solely as a descriptive, hypothesis-generating observation to inform future studies.

Small sample size: Electron microscopy was performed on only 24 donors (18 SIH, 6 NG), which is insufficient for robust statistical correlation with clinical outcomes.

Absence of quantification: The findings were qualitative (e.g., mitochondrial swelling in a minority of cells) and were not systematically quantified (e.g., by morphometric analysis), precluding meaningful statistical testing.

Whether these ultrastructural observations correlate with donor glucose levels, graft function, or clinical outcomes remains unknown, as no correlation analyses were performed due to the exploratory nature of this analysis. Future studies incorporating systematic histopathological quantification and larger sample sizes, along with integrated clinical data, are needed to determine whether such changes have functional significance.

Figure. Histopathological and ultrastructural analysis of donor pancreatic grafts via time-zero biopsy.

(Top panels) Representative hematoxylin and eosin (H&E)-stained sections (100× magnification) from a normoglycemic donor (A) and a donor with stress-induced hyperglycemia (B). Both show preserved pancreatic histoarchitecture with intact acinar cells containing zymogen granules and normal-appearing islets of Langerhans. Scale bar = 100× magnification.

(Bottom panels) Transmission electron microscopy (TEM) images of pancreatic acinar cells from a normoglycemic donor (C) and an SIH donor (D). Scale bar= 2 μm.


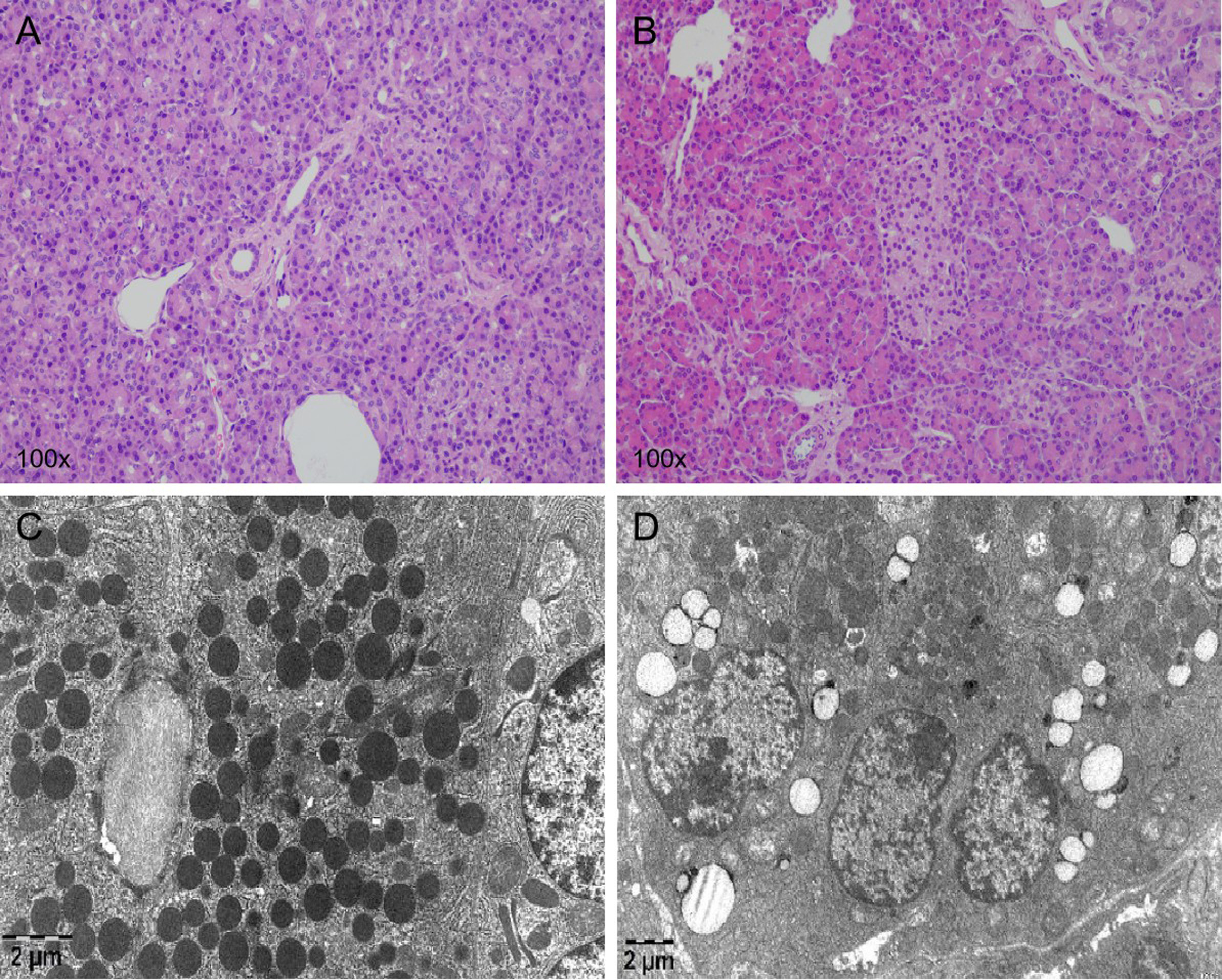

Supplement: Supplementary file 15 [file Table11.doc]
